# Early reduction in aversive Pavlovian bias as a mediator of anhedonia improvement during Behavioural Activation in realistic treatment settings

**DOI:** 10.1371/journal.pcbi.1014439

**Published:** 2026-07-14

**Authors:** Lioba C. S. Berndt, Daisy Crawley, Ruslana Tymchyk, Anna Hall, Jakub Onysk, Tore Erdmann, Elliott Wimmer, Isabel M. Berwian, Agnes Norbury, Quentin J. M. Huys

**Affiliations:** 1 Applied Computational Psychiatry Lab, Mental Health Neuroscience Department, Division of Psychiatry and Max Planck UCL Centre for Computational Psychiatry and Ageing Research, Department of Imaging Neuroscience, Queen Square Institute of Neurology, University College London, London, United Kingdom; 2 North London NHS Foundation Trust, London, United Kingdom; 3 Doctorate in Clinical Psychology, Division of Psychology and Language Sciences, University College London, London, United Kingdom; 4 Max Planck UCL Centre for Computational Psychiatry and Ageing Research, Department of Imaging Neuroscience, Queen Square Institute of Neurology, University College London, London, United Kingdom; 5 Research Department of Clinical, Educational and Health Psychology, University College London, London, United Kingdom; Indian Institute of Technology Mandi - Kamand Campus: Indian Institute of Technology Mandi, INDIA

## Abstract

**Background:**

Behavioral Activation therapy is an effective treatment for major depressive disorder. Conceptually, the mechanisms through which it acts are thought to involve alterations to reinforcement learning, and several recent studies have supported this in laboratory settings. However, it remains unclear whether reinforcement learning mechanisms are involved in a realistic treatment setting.

**Methods:**

In a randomized controlled observational study in the UK NHS talking Therapy service, 152 participants with low mood received reinforcement learning assessments using an affective Go/No-Go task. The assessment timepoints were randomized between subjects, with assessments occurring either before (control; n = 78) or during BA therapy (active; n = 74). Changes in Pavlovian biases were quantified using computational modeling.

**Results:**

Anhedonia improved during treatment, but not before (*p* = 0.047; *d* = 0.53). The active treatment group showed significant changes in Pavlovian parameters compared to controls (*p*_FDR_ = 0.012; *d* = 0.65). Pavlovian biases became more positive in the active group (*M* = 0.44, *SD* = 0.81) and more negative in the control group (M=−0.12, *SD* = 0.70). Changes in aversive Pavlovian bias statistically mediated treatment effects on symptoms of anhedonia (β=−1.77, *p* = 0.036, bootstrap; p = 0.0280).

**Conclusions:**

Behavioral Activation modified Pavlovian biases early in treatment by simultaneously strengthening appetitive and diminishing aversive responses. Reduction in aversive bias statistically mediated improvements in anhedonia, suggesting a specific cognitive mechanism through which Behavioral Activation exerts its therapeutic effects. The study provides evidence that online measurements of cognitive function may have potential as mechanistic biomarkers.

## Author summary

Depression often reduces motivation and the ability to enjoy activities(anhedonia). Behavioral Activation (BA) is a widely used therapy that encourages people to re-engage with meaningful and rewarding activities. While BA is effective, the cognitive processes through which it improves symptoms are not fully understood. One possibility is that BA changes how people automatically respond to potential rewarding and negative outcomes. These automatic tendencies, Pavlovian biases, can make people more likely to act when a reward is expected and more likely to withhold action when a negative outcome is anticipated. In this study, 152 people receiving treatment for low mood in the UK NHS Talking Therapies completed a computer-based learning task either before starting BA or during early therapy. Using computational modelling, we measured changes in Pavlovian biases. Participants assessed during therapy showed a reduction in aversive Pavlovian bias, meaning that potential negative outcomes were less likely to automatically discourage action. Statistical analysis showed that this reduction mediated improvements in anhedonia, linking the cognitive change directly to symptom improvement. These findings suggest that BA helps reduce depressive symptoms partly by altering how people respond to potential negative outcomes, and that simple online tasks could provide measurable markers of this therapeutic mechanism.

For the purpose of Open Access, the author has applied a Creative Commons Attribution (CC BY) public copyright licence to any Author Accepted Manuscript version arising from this submission.

## 1. Introduction

Behavioral Activation (BA) is a key psychotherapeutic treatment for depression, demonstrating effectiveness comparable to Cognitive Behavioral Therapy while offering greater scalability due to its relative simplicity and reduced therapist training requirements [1,2]. Meta-analyses show moderate treatment effects (*d* = 0.34), with particular success in addressing anhedonia, which is otherwise a predictor of poor treatment response [3]. However, remission rates of 56% imply that many do not achieve remission through BA. A better understanding of the underlying cognitive and neurobiological processes that are engaged by behavioral activation could help improve and target treatment better [4–7].

One path towards such an understanding might involve reinforcement learning. Reinforcement learning [8] is a theory of how to learn from (delayed) rewards and losses. The conceptual groundwork of cognitive-behavioral psychotherapy aligns closely with reinforcement learning [9–12], and there is meta-analytic evidence for reinforcement learning deficits in depression [13]. Research on reinforcement learning in humans has yielded substantial advances in our understanding of its cognitive, neural and behavioral structure, meaning that it provides a robust framework for linking aspects of depression to their neurobiological underpinnings and to treatment mechanisms [14]. Researchers have indeed started to use cognitive tasks to more formally test whether and how reinforcement learning processes are engaged by psychotherapy broadly, and by behavioural activation interventions more specifically. Reinforcement learning processes appear to change with psychotherapy [15,16], suggesting that successful psychotherapy may engage reinforcement learning processes. More targeted studies have also attempted to dissect specific processes, and have suggested that different interventions can engage different processes [17,18].

One reinforcement learning component of particular interest are Pavlovian biases [19]. Briefly, Pavlovian biases capture how expectations of rewards lead to action invigoration, while expectations of punishments lead to inhibition of action. Such Pavlovian biases are robustly measurable in humans and interfere with instrumental (goal-directed) decision-making [20–22]. A core component of behavioral activation therapy is to enable patients to act according to pre-specified plans rather than according to momentary emotional experience. This speaks directly to altered Pavlovian processes in depression [23,24], where momentary emotional experiences often direct individuals towards withdrawal and inaction; or fail to energise them towards rewarding experiences. As such, BA therapy at its core involves a strengthening of maladaptively low appetitive Pavlovian biases, and a reduction of maladaptively high aversive Pavlovian biases. However, a direct test of this is still outstanding, with only one small pilot study in a clinical sample receiving behavioral activation reporting that symptom improvement covaried with specific changes in Pavlovian biases [25].

Here, we therefore asked whether Pavlovian biases are engaged by behavioral activation therapy for depression early on. We focused on two time points: baseline (before starting therapy) and three weeks into therapy (assessment time-point 1). By examining these early time points, we investigated whether early changes in Pavlovian biases related to early treatment response. We focused on a naturalistic yet highly controlled treatment setting, namely NHS Talking Therapies [26,27], a UK national treatment service accessible through self-referral or clinician referral. BA was delivered as a low-intensity intervention (Step 2) by accredited Psychological Wellbeing Practitioners (PWPs) following the National Institute for Health and Care Excellence (NICE) guideline for the treatment and management of depression in adults [28]. This delivery format has been validated in large-scale randomised controlled trials [2] and in routine NHS services through IAPT evaluations demonstrating effectiveness and scalability [26]. It is recommended in UK clinical practice for its scalability and cost-effectiveness [28]. Treatment was manualised, and PWPs received accredited training and regular supervision to ensure adherence to the intervention protocol. The study design involved randomly assigning individuals to different assessment time-points (either before therapy had started, or once therapy had started), without affecting either the therapy start date or any other aspect of the therapy itself. We hypothesized *a priori* that behavioral activation would result in a) increases in appetitive and decreases in aversive Pavlovian biases; and b) increases in overall activation (go bias). Furthermore, we hypothesized that changes in Pavlovian biases would be associated with clinical outcomes, namely that an c) increase in appetitive Pavlovian bias would negatively predict depressive symptom severity changes, while d) increase in aversive Pavlovian bias would predict an increase in depressive symptoms. Three of the predictions were confirmed for symptoms of anhedonia, where we found a statistical mediation of the effects of behavioral activation by changes in aversive Pavlovian bias.

## 2. Methods

### 2.1. Ethics statement

The study procedures were approved by the East Midlands - Leicester South Research Ethics Committee (REC reference 20/EM/0219, protocol 125966). All participants provided written informed consent electronically prior to data acquisition.

### 2.2. Setting

The study was delivered within participating NHS Talking Therapies centres in London. NHS Talking Therapies is a UK-wide service providing evidence-based, standardised and manualised psychological therapies for adults with common mental health problems [27,26]. Step 2 treatment for depression in NHS Talking Therapies consists of 3–6 weeks of 1-hour weekly manualised behavioural activation, delivered by accredited PWPs in accordance with the NICE guideline for the treatment and management of depression in adults [28]. In principle, Step 2 BA can be followed by up to 3 weeks of cognitive restructuring, although this is rarely implemented in practice. PWPs receive accredited training and regular supervision to ensure fidelity to the intervention protocol. Additional details on the BA intervention structure and its evidence base are provided in section 4.2 in S1 Appendix.

### 2.3. Eligibility criteria

Participants were eligible for inclusion if they were aged 18–65 years, on the waiting list for Step 2 treatment for depression in one of the participating NHS Talking Therapy service, and able to understand study instructions. They needed to provide informed consent, communicate via email, and access study materials online. Exclusion criteria included having a confirmed IAPT psychotherapy start date within 3 weeks, a current or past history of serious neurological disorders or trauma, or any medical condition or treatment that could pose a risk for participation. Those with a learning disability requiring specialist educational support, inability to understand written and spoken English, or individuals in prison or on probation were also excluded from the study.

### 2.4. Study design

We employed a naturalistic observational study with randomized assessment time-points. Participants were randomized 1:1 to either undergo assessments during their BA therapy (active group) or before starting therapy (control group). Randomization was performed by EW according to a simple block randomization scheme; EW had no other involvement in the study until the analysis and write-up stage. Randomization only affected assessment timing and did not alter planned clinical care or therapy start dates.

The active group assessment schedule included data collection one week prior to therapy start (baseline), followed by three assessment points during the therapy: session 1 (at week 3 of therapy), session 2 (at week 6, upon completion of the first therapeutic module), and session 3 (at week 9, marking the end of their therapy). The control group was scheduled for a corresponding baseline assessment within one week of enrollment, followed by three weekly assessment timepoints prior to starting BA therapy. Both groups completed follow-up questionnaires six weeks after therapy completion. A schematic overview of the full assessment schedule is provided in S1 Appendix.

The study aimed to recruit 145 participants, targeting a final sample of 126 participants (63 per group) after accounting for an anticipated 20% dropout rate. The sample size was determined by a power analysis for a two-sample t-test, assuming an effect size (Cohen’s d) of 0.5, an alpha level of .05, 80% power, and a 1:1 allocation ratio.

### 2.5. Procedure

Individuals who had been accepted for Step 2 depression intervention and were waiting for their therapy to start were screened. Following expressions of interest, contact details were provided to the research team. The research team sent the Patient Information Sheet electronically and contacted potential participants to address any queries. Participants provided informed consent electronically. Communication with participants was conducted via email, and participants performed all study activities online through the browser. The main assessments included a battery of tasks and self-report questionnaires. Participants could interrupt the assessment between tasks and questionnaire batches, but were required to complete each battery within a 7-day window from receiving each assessment link. The full battery took 130 minutes per time point.

#### 2.5.1. Self-report questionnaires.

The battery included measures of depression (Patient Health Questionnaire [PHQ-9] [29], Inventory of Depressive Symptomatology [IDS-SR30] [30]), anxiety (Generalized Anxiety Disorder Scale [GAD-7] [31]), and pleasure experience (Temporary Experience of Pleasure Scale [TEPS] [32]). Additional measures assessed cognitive patterns (Ruminative Response Scale [RSQ] [33], Beck Dysfunctional Attitude Scale [DAS] [34]) and therapeutic engagement (Working Alliance Inventory [WAI] [35], Behavioural Activation for Depression Scale Short Form [BADS] [36]). The study was conducted within NHS Talking Therapies, where PHQ-9 administration is routine practice. A safeguarding protocol was in place: Endorsement of suicidal ideation triggered immediate review by the study’s clinical lead (psychiatrist) and signposting or referral to appropriate support services.

#### 2.5.2. Affective Go/No-Go Task.

**Fig 1:**
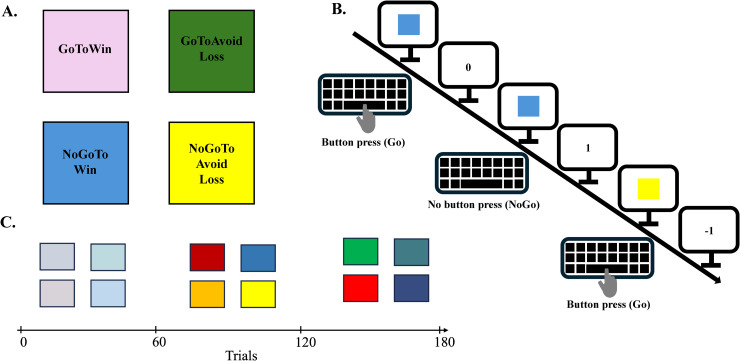
Affective Go/No-Go Task. **A.** The task conditions consisted of four combinations of response type (Go/NoGo) and outcome valence (Win/Avoid Loss), forming the four experimental conditions. In this figure, colors are used to represent these different conditions and indicate the corresponding stimulus types; in the actual task, participants saw distinct stimuli (e.g., boat, bike, etc.) - not colors - for each condition. **B.** Trial structure and feedback system displaying point outcomes (0, 1, -1) for Go (button press) and NoGo (no button press) responses. Correct actions in win conditions yielded rewards, while correct actions in avoid-loss conditions prevented losses. **C.** Task structure included three 60-trial blocks, totaling 180 trials per session. Different colors between trial-blocks represent four different stimulus types used in this round.

Participants performed an adapted version of the affective Go/No-Go task [20] which measures participants’ decisions to either take an action (Go) or withhold an action (NoGo) in response to reward and punishment cues. The task consisted of four conditions: GoToWin, GoToAvoidLoss, NoGoToWin, and NoGoToAvoidLoss (Fig 1A). In each trial, participants saw one of four unique images and had to learn whether to press a button (Go) or withhold their response (NoGo).

Correct responses in “win” conditions (GoToWin and NoGoToWin) resulted in one point gained, while correct responses in “avoid loss” conditions (GoToAvoidLoss and NoGoToAvoidLoss) prevented losing a point. Incorrect responses either forfeited a reward or resulted in a loss (Fig 1B).

The adapted task included several modifications to improve test-retest reliability and informativeness [37,38]. (1) Feedback was probabilistic: in 90% of trials, feedback was congruent with the image’s category, while the remaining 10% introduced incongruent feedback to maintain uncertainty. (2) The task was structured into three rounds per session, with 180 trials divided into three 60-trial blocks (Fig 1C). This increased the number of trials per session, providing a larger dataset and preventing participants from achieving perfect learning. (3) To maximize comparability between participants, stimulus sequences were pseudorandomized, with all participants seeing the same sequence. Blocks and sessions contained different sequences.

Results for the other tasks in the battery will be reported elsewhere.

### 2.6. Analyses

#### 2.6.1. Missing data.

The present report focuses on early changes between baseline and session 1. Baseline characteristics of completers versus non-completers were compared (Table A in S1 Appendix). All analyses were initially conducted on a complete-case (per-protocol), unless explicitly stated otherwise. To assess the robustness of the findings, two additional analyses were performed: a modified intention-to-treat (mITT) including all participants with baseline data; and an intention to treat (ITT) analysis including all randomised participants after multiple imputation with chained equations (MICE; section 4.10 in S1 Appendix).

#### 2.6.2. Changes in clinical symptoms.

We tested for normality using the Lilliefors test. Symptom changes were examined using dependent t-tests for within-group comparisons and independent t-tests for between-group comparisons. When the assumption of normality was violated, we employed the Wilcoxon signed-rank test for within-group comparisons and the Mann-Whitney U test for between-group comparisons.

#### 2.6.3. Linear mixed effects analyses.

To analyze task performance dynamics over time, we modeled overall accuracy using a mixed-effects model (section 4.6 in S1 Appendix). The linear mixed-effects model was initially conducted on all participants with at least baseline data (mITT).

#### 2.6.4. Computational models.

We examined a series of 5 nested reinforcement learning models of increasing complexity, guided by previously published models [20,37,39,40].

All models computed action probabilities based on action weights $W(a_{t},s_{t})$, transformed using a softmax function [41]


$$\begin{array}{c} p(a_{t}|s_{t})=\left[\frac{\exp(W(a_{t}|s_{t}))}{\sum_{a^{\prime }}\exp(W(a^{\prime }|s_{t}))}\right](1-\xi )+\frac{\xi }{2} \end{array}$$ (1)

where ξ represents irreducible noise (constrained between 0 and 1).

In the base model, action weights were just the instrumental *Q* values, i.e.,


$$\begin{array}{c} W_{t}(a_{t},s_{t})=Q_{t}(a_{t},s_{t}) \end{array}$$

This was retained for “nogo” actions in all subsequent models.

The *Q*-values were updated using a standard Rescorla-Wagner model [42]:


$$\begin{array}{c} Q_{t}(a_{t},s_{t})=Q_{t-1}(a_{t},s_{t})+\epsilon \left(\rho r_{t}-Q_{t-1}(a_{t},s_{t})\right) \end{array}$$ (2)

where ϵ is the learning rate, controlling the speed of value updating and ρ is the reward sensitivity, scaling the impact of received rewards ($r_{t}$).

The first extension included a **go-bias** added to “go” action to capture overall biases towards or against emitting active go actions:


$$\begin{array}{c} W_{t}(a_{t},s_{t})=\left\{ \begin{array}{cc} Q_{t}(a_{t},s_{t})+b & \text{if }a_{t}\text{ is go action},\\ Q_{t}(a_{t},s_{t}) & \text{if }a_{t}\text{ is nogo action}. \end{array}\right. \end{array}$$

Next, a **Pavlovian bias** (π) was added to capture a tendency to emit more go actions for positive, and more nogo actions for negative stimuli:


$$\begin{array}{c} W_{t}(a_{t},s_{t})=\left\{ \begin{array}{cc} Q_{t}(a_{t},s_{t})+b+\pi V_{t}(s_{t}), & \text{if }a_{t}\text{ is the go action},\\ Q_{t}(a_{t},s_{t}), & \text{if }a_{t}\text{ is the no-go action}. \end{array}\right. \end{array}$$

where stimulus values ($V_{t}(s_{t})$) were updated using a similar Rescorla-Wagner rule:


$$\begin{array}{c} V_{t}(s_{t})=V_{t-1}(s_{t})+\epsilon \left(\rho r_{t}-V_{t-1}(s_{t})\right) \end{array}$$ (3)

Hence, a positive stimulus value $V_{t}$ increases the tendency towards a go action, while a negative value reduces it, and thereby increases the tendency to nogo.

The next allowed the Pavlovian bias parameter π to differ according to whether the stimulus predicted rewards or losses (and hence whether *V* was positive or negative):


$$\begin{array}{c} W_{t}(a_{t},s_{t})=\left\{ \begin{array}{cc} Q_{t}(a_{t},s_{t})+b+\pi_{\text{app}}V_{t}(s_{t}), & \text{if }a_{t}\text{ is go action and }V_{t}(s_{t})>0,\\ Q_{t}(a_{t},s_{t})+b+\pi_{\text{av}}V_{t}(s_{t}), & \text{if }a_{t}\text{ is go action and }V_{t}(s_{t})<0,\\ Q_{t}(a_{t},s_{t}), & \text{if }a_{t}\text{ is nogo action.} \end{array}\right. \end{array}$$

Finally, an **initial** *Q*_**0**_**-value** was introduced for all state-action pairs:


$$\begin{array}{c} Q_{0}(go,s)=Q_{\text{init}} \end{array}$$

where *Q*_init_ represents the starting value of *Q*-values, reflecting participants’ prior expectations before learning begins.

#### 2.6.5. Change models.

These individual session models were extended into change models to capture between-session differences.

Each change model assumed that one parameter changed between sessions:


$$\begin{array}{c} \theta_{\text{session 1}}=\theta_{\text{baseline}}+\Delta \theta \end{array}$$ (4)

while the other parameters remained fixed. We constructed separate change models in which each parameter was allowed to vary between sessions while all other parameters were held constant. Thus, for each parameter, a corresponding change model estimated a baseline value and a session-related shift. For the Pavlovian bias change model, the session shift was implemented as a single additional parameter inducing a coupled change in appetitive and aversive Pavlovian bias. Specifically, this parameter increased the appetitive bias while decreasing the aversive bias by the same magnitude, thereby enforcing opposing changes rather than allowing them to vary independently.

#### 2.6.6. Model fitting and comparison.

We used a hierarchical Bayesian approach to estimate model parameters and to compare models [21]. Individual maximum a posteriori parameter estimates were extracted for each parameter, participant and time-point. Parameters were transformed prior to estimation to enforce constraints. The fitting procedure was validated using synthetic data to confirm the reliability of parameter recovery. Approximate Bayesian model comparison was performed using the integrated Bayesian Information Criterion (iBIC; [21]).

### 2.7. Therapy-induced changes in computational parameters

To examine therapy-induced changes in computational parameters, we first identified the most parsimonious model using baseline data from both groups. Change models were then fitted to each subject’s combined baseline and session 1 data, assuming all participants are drawn from one group (i.e., enforcing a single joint prior distribution).

Change parameters (Δθ) between the active treatment and control groups were then compared using independent samples t-tests, with Welch’s correction for unequal variances where appropriate. Hence, the model fitting with a single prior assumes a single distribution (no group differences), and the t-test tests for a violation of this null hypothesis. Multiple comparison correction was implemented using the False Discovery Rate (FDR) method across our primary hypotheses.

Our main hypothesis of interest related to a simultaneous change in two parameters (both an increase in the appetitive Pavlovian and a decrease in the aversive Pavlovian bias). To capture this, we examined $\Delta \pi_{\text{combined}}$ in a model where this was both *added* to $\pi_{\text{app}}$ and *subtracted* from $\pi_{\text{av}}$. We also examined changes across sessions in go bias *b*, and the separate appetitive Pavlovian bias $\pi_{\text{app}}$, and aversive Pavlovian bias $\pi_{\text{av}}$. Exploratory analyses examined changes in all remaining model parameters and applied FDR correction across all tested parameters.

To directly link the model-derived $\Delta_{combined}$ effect to observable behaviour, post-hoc Wilcoxon signed-rank tests were conducted in Active group completers (n = 23), comparing % Go responses between Baseline and Session 1 for the two incongruent conditions (NoGo-to-Win and Go-to-Avoid-Loss), where Pavlovian bias competes with the correct instrumental response (see section 4.6 in S1 Appendix).

When then investigated whether changes in computational parameters Δθ related to clinical improvement by performing linear regression analyses. For parameters that emerged as significant predictors of clinical outcomes, we conducted mediation analyses to examine whether they explained the effect of treatment on symptom changes. We first tested whether treatment group predicted symptom changes from baseline to session 1 (total effect), and then examined whether this relationship remained significant after accounting for changes in computational parameters (direct effect), while testing whether the computational parameters themselves were associated with symptom changes in this model (mediation effect; [43]).

## 3. Results

### 3.1. Participant characteristics and study flow

**Fig 2:**
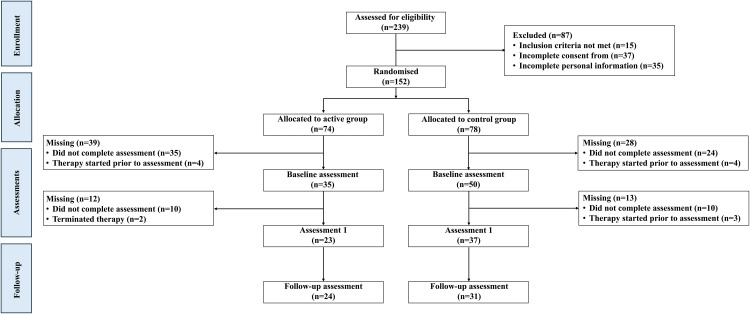
CONSORT flow diagram of participant recruitment, allocation, and follow-up. The diagram illustrates the flow of participants from eligibility assessment (n = 239) through randomization (n = 152) into the active group (n = 74) and control group (n = 78). It details the number of participants completing assessments at baseline (Active: n = 35, Control: n = 50), Assessment 1 (Active: n = 23, Control: n = 37), and the final 6-week post-therapy follow-up (Active: n = 24, Control: n = 31). Reasons for exclusion and participant withdrawal or loss to follow-up at each stage are summarized.

A total of 239 individuals were assessed; 152 were randomized (Active: 74, Control: 78) after exclusions (n = 15 ineligible, n = 37 incomplete consent, n = 35 incomplete personal information). Baseline demographic and clinical characteristics were similar between groups (Table 1). Of those randomized, 35 Active and 50 Control participants completed baseline. For the primary early-change analyses (baseline to assessment 1), data were available from 23 Active and 37 Control participants; at the 6-week post-therapy follow-up, 24 Active and 31 Control participants provided self-report data (Fig 2). A schematic of the full planned assessment schedule is provided in the supplements (Fig A in S1 Appendix). Analyses focus on early changes between baseline to session 1, and on the relationship of these changes to post-therapy follow-up. We perfomed complete-case-analyses, a modified intention-to-treat (mITT) including all participants with baseline data; and an intention to treat (ITT) analysis including all randomised participants after multiple imputation with chained equations (MICE; section 4.10 in S1 Appendix). Unless stated otherwise, analyses refer to the complete-case (per-protocol) sample.

**Table 1:** Participant demographics. Groups were well-matched. Percentages represent the proportion of participants within each group. The p-values are for chi-square tests.

Category	Active group	Control group	p-value
Age	Mean: 32.55 (±8.21)	Mean: 33.35 (±8.99)	0.708
Gender	Female: 72.06% Male: 26.47%, Non-binary: 1.47%	Female: 61.04%, Male: 36.36%, Non-binary: 2.60%	0.249
Sex	Female: 72.06%, Male: 27.94%	Female: 62.34%, Male: 37.66%	0.189
Ethnicity	White: 52.94%, Asian: 19.12%, Mixed: 8.82%, Black: 13.24%, Other: 5.88%	White: 53.25%, Asian: 18.18%, Mixed: 11.69%, Black: 6.49%, Other: 10.39%	0.409
Employment	Employed: 48.53%, Full-time Education: 22.06%, Recently Unemployed: 17.65%, Unemployed: 2.94%, Other: 7.35%, Unknown: 1.47%	Employed: 53.25%, Full-time Education: 19.48%, Recently Unemployed: 15.58%, Unemployed: 7.79%, Other: 3.90%, Unknown: 0.00%	0.388
Medication Status	On Medication: 57.35%, No Medication: 42.65%	On Medication: 53.25%, No Medication: 46.75%	0.659

### 3.2. Behavioral activation improves anhedonia

**Fig 3:**
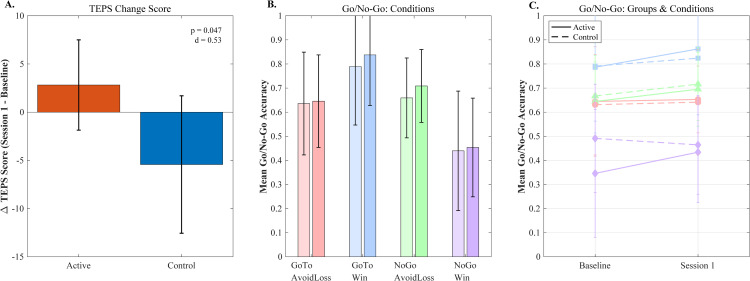
Behavioural activation effects on TEPS change scores and Go/No-Go task accuracy. **A.** Mean (±SD) change in TEPS score from Baseline to Session 1. P-value and Cohen’s d from an independent samples comparison are shown. **B.** Mean (±SD) Go/No-Go accuracy by condition, averaged across groups. Lighter bars represent Baseline, darker bars represent Session 1. **C** Mean (±SD) Go/No-Go accuracy from baseline to session 1, plotted separately for Active (solid lines) and Control (dashed lines) groups across the four conditions (colors/markers distinguish conditions).

Both groups showed significant IDS (Active: M = -4.51, SD = 7.23, p = 0.016; Control: M = -3.05, SD = 4.75, p = 0.018) and PHQ9 reductions (Active: M = -1.69, SD = 2.66, p = 0.024; Control: M = -1.95, SD = 2.35, p = 0.002) at assessment 1. Group differences were non-significant for IDS (p = 0.441) and PHQ9 (p = 0.902). TEPS scores improved in the Active group (M=+2.82, SD = 4.68) but worsened in Control (M = -5.43, SD = 7.13), a significant difference (p = 0.047, d = 0.53; Fig 3A). This difference was absent at 6-week follow-up after both groups completed BA, with similar positive changes (p = 0.375, d = 0.30; Fig E). No other questionnaires changed significantly; full results are in the supplements (section 4.5 in S1 Appendix).

### 3.3. Go/No-Go task performance varies by condition

A mixed-effects analysis of Go/No-Go accuracy revealed a main effect of condition (Fig 3B): accuracy was higher for GoToWin (β=0.142, p = 0.024) but lower for NoGoToWin (β=−0.298, p < .001) vs. GoToAvoidLoss. No significant group, time, or interaction effects emerged. Age and sex covariates were non-significant.

### 3.4. Computational analyses

To understand the learning and affective changes driving accuracy changes, we examined computational models capturing trial-by-trial decision-making trajectories.

**Fig 4:**
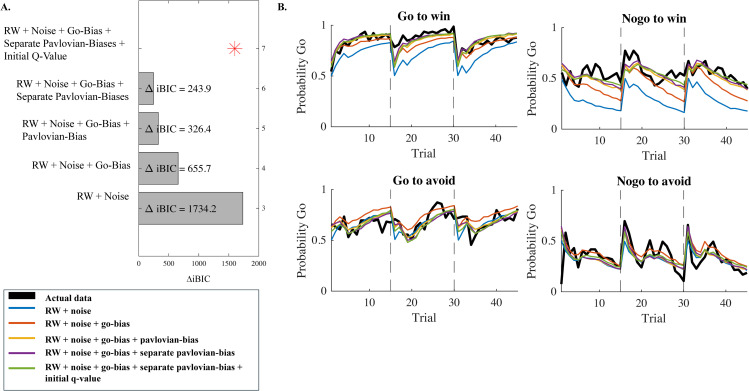
Simulated learning curves across models and conditions. **A.** Integrated Bayesian Information Criterion (ΔiBIC) scores for each model, highlighting the trade-off between model complexity and fit. Lower ΔiBIC values indicate better model fit after penalizing complexity. Numbers to the right denote the number of parameters for each model. The red asterisk marks the winning model with the best balance of fit and parsimony. **B.** Panels show the probability of Go responses across 45 trials for four task conditions (*GoToWin*, *NoGoToWin*, *GoToAvoidLoss*, and *NoGoToAvoidLoss*) for different model architectures. The black lines represent actual data, while the colored lines correspond to each model’s predicted probabilities of Go actions. Dashed vertical lines indicate block transitions (15 trials per condition per block). The inclusion of additional model components (e.g., Go-Bias, Pavlovian-Biases, and Initial Q-Value) leads to progressively better fits to the observed learning patterns, particularly in Pavlovian conditions that require overcoming natural tendencies (e.g., *NoGoToWin* or *GoToAvoidLoss*).

Participants’ behavior was most parsimoniously explained by a model incorporating both instrumental learning and Pavlovian influences (Fig 4A). The winning model included seven parameters: a learning rate (ε) for updating action and stimulus values, reward sensitivity (ρ) scaling the impact of outcomes, initial Q-values (*Q*_0_), a Go bias (*b*) capturing general action tendency, separate Pavlovian biases for reward ($\pi_{\text{app}}$) and loss ($\pi_{\text{av}}$) conditions reflecting automatic approach-avoidance influences, and irreducible noise (ξ). Fig 4B shows that the most parsimonous model captured the average behavioural patterns well, and captured it better than simpler models. This model hence measures meaningful Pavlovian biases after correcting for all other processes. To further illustrate the contribution of specific model components, Fig B and Fig C in S1 Appendix show how the inclusion of the initial Q-value parameter improves the model’s ability to capture early response tendencies, and how separate Pavlovian biases for Win and Avoid conditions better reproduce the observed differences in Go responding across conditions. We verified the psychometric properties of the task, showing good internal consistency and task reliability (section 4.8 in S1 Appendix). Furthermore, recovery analyses showed good recoverability for the winning model (section 4.9 in S1 Appendix).

#### 3.4.1. Behavioural Activation Increases Appetitive and Reduces Aversive Pavlovian Biases.

**Fig 5:**
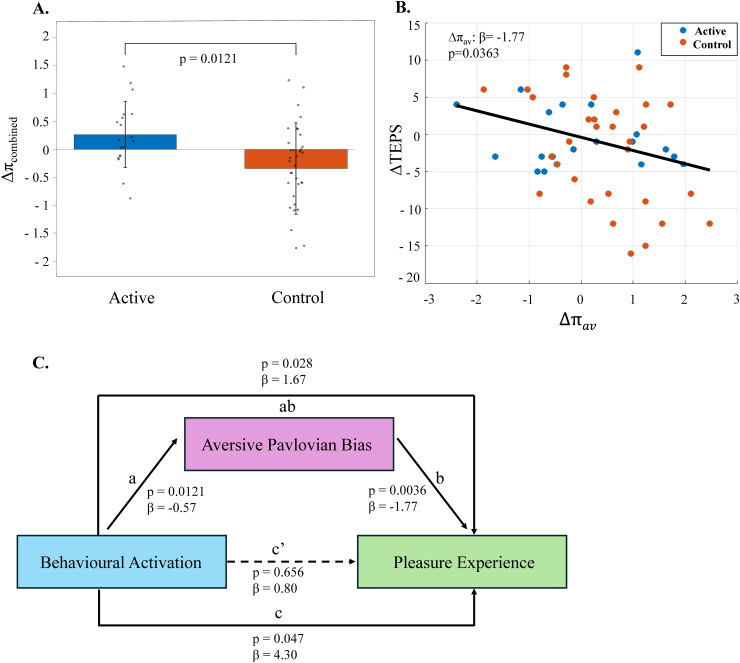
The effect of behavioural activation on anhedonia is mediated by changes in Pavlovian bias. **A.** Comparison of change in combined Pavlovian bias ($\Delta \pi_{\text{combined}}$) between Active and Control groups. Bars represent mean ± SD, grey dots show individual participants. The p-value indicates a significant difference between groups (p = 0.0121). **B.** Relationship between change in aversive Pavlovian bias ($\Delta \pi_{\text{av}}$) and change in TEPS score (ΔTEPS). Points are coloured by group (Active: blue, Control: orange). Regression line is shown for both groups combined. Statistics reflect the significant overall negative association between $\Delta \pi_{\text{av}}$ and ΔTEPS across groups (β=−1.77,p=0.0363). **C.** Mediation path diagram testing whether the effect of Behavioural Activation (Group assignment: Active vs. Control) on Pleasure Experience (ΔTEPS) is mediated by changes in Aversive Pavlovian Bias ($\Delta \pi_{\text{av}}$). Path coefficients (b) and p-values are displayed for the effect of group on the mediator (path **a)**, the mediator on the outcome (path **b)**, the total effect of group on the outcome (path **c)**, and the direct effect of group on the outcome controlling for the mediator (path c’). Bootstrap analysis indicated a significant indirect mediation effect (ab; β=1.67,p=0.028,CI:[0.1334,3.8722]).

Having validated the computational description of task behaviour, we proceeded to examine the parameters quantifying specific processes. In the completer (per-protocol) sample, behavioural activation shifted the balance of Pavlovian biases, enhancing appetitive and reducing aversive Pavlovian biases at session 1 relative to baseline. Groups differed in the combined Pavlovian bias change $\Delta \pi_{\text{combined}}$, and this survived FDR correction for the two main hypotheses (p_FDR_ = 0.012; d = 0.65), with the active treatment group showing a positive change (M = 0.44, SD = 0.81), indicating simultaneously increased appetitive and decreased aversive Pavlovian influences, while the control group showed a negative change (M = -0.12, SD = 0.70; Fig 5A). The effect remained statistically significant in a mITT analysis (active group: positive change in the combined Pavlovian bias (M = 1.00, SD = 0.76); control group: negative change (M = -1.83, SD = 0.82); difference: p_FDR_ = 0.0415) but not in an ITT analysis after imputation (section 4.10). To provide an intuitive behavioural interpretation of these parameter changes, we additionally visualised how systematic variation in the appetitive and aversive Pavlovian bias parameters affects the probability of Go responses across task conditions (Fig G in S1 Appendix).

There was no group difference in go bias change Δb. Exploratory analyses of changes in other model parameters revealed no significant group differences.

#### 3.4.2. Reduction in aversive pavlovian bias is associated with enhanced pleasure experience.

Changes in the combined Pavlovian bias $\Delta \pi_{\text{combined}}$ were positively associated with TEPS changes (β=2.19, *p* = 0.011) from baseline to assessment 1. This was driven by changes in the aversive Pavlovian bias ($\Delta \pi_{\text{av}}$), which were negatively associated with change in anhedonia (TEPS total score; β=−1.77, *p* = 0.0363). This association remained significant (p = 0.041) after including a group*aversive Pavlovian bias interaction (p = 0.063), suggesting the relationship was not specific to either group.

Hence, increased appetitive and decreased aversive Pavlovian bias were associated with enhanced pleasure (Fig 5B). Again, mITT analyses yielded consistent results ($\Delta \pi_{\text{combined}}:\beta =1.45,p=0.039;\;\Delta \pi_{\text{av}}:\beta =-0.89,p=0.047$), while ITT analysis after imputation, showed the same direction, but did not reach significance (section 4.10 in S1 Appendix).

Exploratory complete-case analyses revealed that changes in learning rate (Δε) were negatively associated with changes in PHQ9 scores (β=−0.74, *p* = 0.04) and changes in reward sensitivity were positively associated with changes in dysfunctional beliefs (DAS β=5.74, *p* = 0.024).

All relationships remained significant after controlling for age and sex. No other computational parameters showed significant associations with clinical outcome measures.

#### 3.4.3. Decreases in aversive Pavlovian bias mediate BAs effects on pleasure experience.

Given the associations between aversive Pavlovian bias and pleasure experience, and the opposing TEPS trends between groups (Active: *M* = +2.82, *SD* = 4.68; Control: M=−5.43, *SD* = 7.13; *p* = 0.047), we conducted a mediation analysis. The effect of treatment group on TEPS changes became non-significant (β=0.80, *p* = 0.66) when controlling for $\Delta \pi_{\text{av}}$, while $\Delta \pi_{\text{av}}$ remained significant (β=−1.77, *p* = 0.036; Fig 5C). A bootstrap analysis confirmed the mediation effect (ab;β=1.67, *p* = 0.028). Again, the mITT analysis revealed the same pattern (ab;β=0.93, *p* = 0.034), but ITT after imputation did not (section 4.10 in S1 Appendix).

We also examined whether changes in aversive Pavlovian bias from baseline to session 1 predicted changes in TEPS from session 1 to session 2. This analysis showed a trend in the expected direction (β=−0.82, *p* = 0.059).

Finally, the existence of a mediation effect suggests that those individuals who have the highest Pavlovian aversive inhibition might benefit most from BA. We therefore asked whether baseline measurements of aversive Pavlovian function predict the overall treatment effects at follow-up (week 16). A higher baseline aversive Pavlovian bias ($\pi_{\text{av}}$) was indeed associated with greater increases in TEPS pleasure experience from baseline to follow-up, but this relationship did not reach statistical significance (β=1.078, *p* = 0.126).

## 4. Discussion

This study investigated how Behavioral Activation therapy influences reinforcement learning mechanisms in individuals with major depressive disorder; and whether this is related to symptomatic response. In an attempt to identify therapeutic mechanisms [44], our study combined intervention research in realistic treatment settings with the deployment of online, scalable but precise assessment of cognitive and learning functions using tasks and computational modelling [45,46]. The results indicate that BA therapy, as delivered in the NHS Talking Therapies setting, has an early effect on self-reported symptoms of anhedonia (TEPS; [32]); and modifies both appetitive and aversive Pavlovian biases in opposite direction. Importantly, we found that the effect of the therapy on symptoms was fully mediated by the effect of BA therapy on aversive Pavlovian biases.

The observed changes in Pavlovian biases align closely with the putative underlying active mechanisms of BA [10] and a previous small pilot study [25]. Specifically, BA theory emphasizes the importance of increasing engagement with rewarding activities and reducing or overcoming low mood-driven avoidance of potentially aversive situations. The computational modeling results provide direct, but partial support for both components of this theory. First, they suggest that the intervention does affect both the positive and negative Pavlovian bias; and in opposite direction. This is notable as the model comparison suggested that the positive and negative Pavlovian bias varied separately, requiring two separate parameters for a parsimonious account of the data. Second, improvements in anhedonia were primarily linked to the prevention of an increase, but also to decreases, in aversive Pavlovian bias. Relatedly, a trend-wise Group × Time interaction in accuracy driven by reduced NoGoToWin performance following behavioural activation (Fig H in S1 Appendix) is consistent with the modelling results: increased appetitive Pavlovianian bias promotes action in reward contexts, selectively impairing performance in this instrumentally incongruent condition. This is intriguing and maybe suggests a particularly important component of aversive inhibition in the experience and maintenance of anhedonia rather than a central reduction in the power of appetitive expectations. Our findings are partially in keeping with previous reports [15] whereby a full course of CBT had effects on learning from rewards and losses, and the changes in learning also related to anhedonia. Here, we do not observe changes in the learning rates themselves. However, it should be noted that the Pavlovian parameters in the Go/Nogo task have some similarity to specific learning rates.

Interestingly, both groups showed reductions in global depressive symptom severity over the early assessment window, whereas only anhedonia showed a group-specific effect. The reasons for the shared improvement in global symptoms might have arisen from the relative brevity of the wait, which may have been a (positive) surprise to participants. The group-specific effect on anhedonia is notable: anhedonia is generally considered harder to shift than global depressive symptom severity, so finding differential effects precisely on this outcome suggests BA is exerting a specific early influence on hedonic functioning. This is consistent with prior work identifying anhedonia as a key target of BA [47] and raises the possibility that anhedonia improvements may precede broader symptomatic change, which is more commonly reported at later treatment endpoints in BA trials [2]. The focus of the TEPS used to measure anhedonia is more on momentary hedonic experience which may also make it more sensitive to early differential effects than the PHQ-9 and IDS, which rely on retrospective symptom ratings over a longer period.

Next, we note that anhedonia is a core predictor of poor treatment response and often resistant to conventional interventions [3]. As such, these results might point to the importance of Pavlovian processes in the maintenance of difficult to treat depression, and it might be worthwhile considering these as treatment targets in their own right [48,49]. It also raises the possibility that BA may work in part by directly targeting the automatic aversive avoidance and blunted appetitive motivation that characterise Pavlovian biases in depression, encouraging approach behaviour in contexts where avoidance has become the default, and in doing so restoring more normal patterns of hedonic engagement. This would suggest that individuals with stronger aversive Pavlovian bias at baseline may benefit most from BA, motivating future work to test this as a predictor of treatment response and a marker for treatment stratification.

One notable outcome is the successful translation of laboratory-based findings into a real-world clinical setting. This is challenging due to varying patient engagement [50], task acceptability, and test-retest validity concerns [38]. Similar versions of this task had shown small effects in larger samples due to poor reliability [38,51]. Here, by shortening the main component and repeating it over multiple blocks, we achieved acceptable test-retest reliability. This allowed us to identify correlations with symptom changes in our sample, despite its size and real-world setting [52]. The fact that these changes are observed in routine clinical practice, with therapy delivered by various therapists and assessments done online, attests to the viability and scalability of such methods [22].

### 4.1. Limitations

The high attrition rate during treatment limited both our ability to examine longer-term maintenance of effects and the statistical power and precision of early (3-week) analyses. Consequently, null effects on symptom measures should be interpreted as inconclusive rather than evidence of absence, and the magnitude of statistically significant early effects may be overestimated (Type-M error; [53]). Most early effects of interest were in the small-to-moderate range, which is difficult to detect reliably with the available sample. Replication in larger samples with improved retention will be necessary to confirm these early mechanistic findings. Moreover, as the mediator (Pavlovian bias change) and outcome (anhedonia change) were measured over the same time window, temporal precedence cannot be established and no causal inference can be drawn. Future studies should address these issues by improving task acceptability and retention. Finally, this was a per-protocol rather than intention-to-treat analysis, allowing us to focus on mechanisms in those who received the intervention but limiting generalisability.

## 5. Conclusion

This study provides evidence that BA therapy engages reinforcement learning mechanisms in a real-world clinical setting. BA modifies Pavlovian biases early in treatment by simultaneously strengthening appetitive and diminishing aversive responses. Most importantly, a reduction in aversive Pavlovian bias mediates improvements in anhedonia, identifying a key mechanism through which BA exerts its therapeutic effects. The findings underscore the value of computational approaches in elucidating the cognitive processes underlying therapeutic change. Future studies should aim to replicate these findings in larger samples with more balanced retention and investigate whether these early Pavlovian bias changes can predict individual treatment outcomes in the longer run.

## Supporting information

S1 Appendix Contains: complete CONSORT flow diagram (Fig A); baseline characteristics of completers vs. non-completers (Table A); description of NHS Talking Therapies and behavioural activation delivery; model performance analyses including the contribution of the initial Q-value parameter (Fig B) and separate Pavlovian bias parameters for Win/Avoid conditions (Fig C); questionnaire score summaries across groups (Fig D); changes in TEPS scores from baseline to 6-week follow-up (Fig E); Go/No-Go task accuracy main and interaction effects from linear mixed-effects modelling (Fig F); trial-by-trial Go responses as a function of Pavlovian bias parameters (Fig G); task reliability analyses across split methods (Fig H); parameter recovery analyses (Fig I); and intention-to-treat analyses on multiply imputed data. (PDF)
