# Supplementary material for: Early reduction in aversive Pavlovian bias as a mediator of anhedonia improvement during Behavioural Activation in realistic treatment settings
Source: PLoS Comput Biol. 2026 Jul 14;22(7):e1014439. doi: 10.1371/journal.pcbi.1014439 (PMC13387611; doi:10.1371/journal.pcbi.1014439)
Supplement: S1 Appendix — (PDF) [file pcbi.1014439.s001.pdf]

S1 Appendix

4.1 Additional Information: Participant Flow

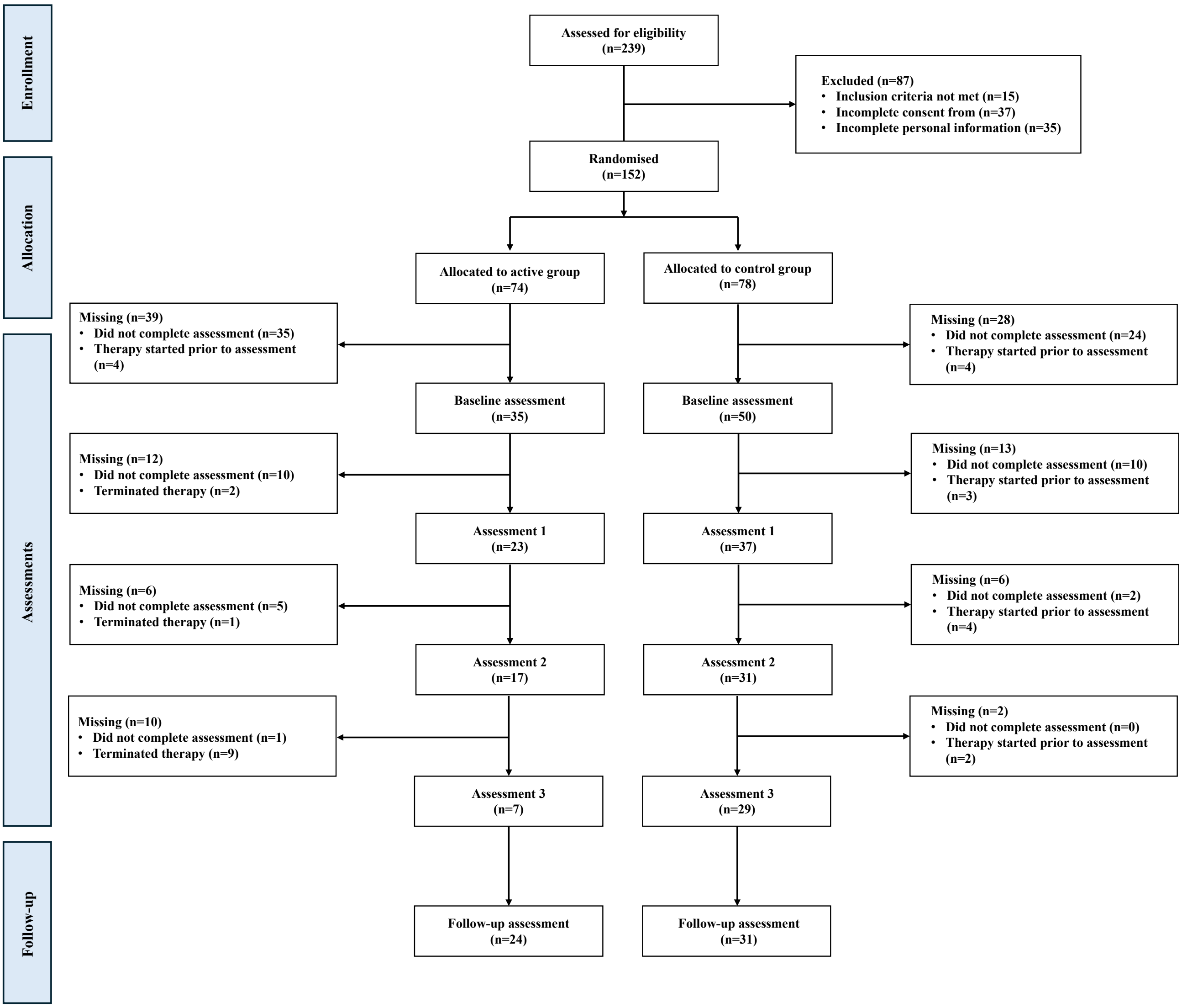

Fig A: ***Complete CONSORT flow diagram of participant recruitment, allocation, and follow-up.*** The diagram illustrates the flow of participants from eligibility assessment (n=239) through randomization (n=152) into the active group (n=74) and control group (n=78). It details the number of participants completing assessments at baseline, assessment 1, assessment 2, assessment 3, and the final 6-week post-therapy follow-up. Reasons for exclusion and participant withdrawal or loss to follow-up at each stage are summarized.

A total of 239 individuals were initially assessed for eligibility in this study. From this pool, 87 individuals were excluded: 15 did not meet the specified inclusion criteria, 37 provided incomplete consent, and 35 had incomplete personal information. Consequently, 152 participants were randomized into the study arms.

The active group was allocated 74 participants. At the baseline assessment, 39 of these participants were missing. This was primarily because 35 did not complete the assessment, and 4 were not assessed because they had initiated therapy before the scheduled baseline assessment, which would have confounded the baseline measures. This left 35 participants who completed this stage. For Assessment 1, an additional 12 participants were lost: 10 did not complete the assessment, and 2 were no longer included as they had terminated therapy, making further on-protocol assessment points irrelevant. This resulted in 23 participants. At Assessment 2, further 6 participants were missing: 5 did not complete the assessment, and 1 had terminated therapy. This left 17 participants. Before Assessment 3, another 10 participants were lost: 1 did not complete the assessment, and 9 had terminated therapy. This resulted in 7 participants. At the final follow-up assessment, data were available for 24 participants from the active group.

The control group received an allocation of 78 participants. During the baseline assessment, 28 participants were missing. Of

these, 24 did not complete the assessment, and 4 were not assessed as they had started therapy prior to the baseline visit, which would have compromised the integrity of the initial, pre-intervention data. This left 50 participants completing baseline. For Assessment 1, an additional 13 participants were unavailable: 10 did not complete the assessment, and 3 were not assessed as they had begun therapy before this assessment point. This left 37 participants. At Assessment 2, another 6 participants were missing: 2 did not complete the assessment, and 4 were not assessed due to starting therapy prior to this time point. This resulted in 31 participants. Prior to Assessment 3, 2 more participants were lost as they had started therapy before this assessment, leaving 29 participants. The follow-up assessment for the control group included data from 31 participants.

Across participant/timepoints, mean total completion time was 97 min (SD = 16), and 73% completed all tasks on the same calendar day.

Baseline characteristics did not differ significantly between completers and non-completers (all $p > 0.05$).

**Category**	**Completers**	**Non-completers**	**p-value**
Age (years)	Mean: 33.1 (±8.5)	Mean: 32.8 (±8.9)	0.821
Gender	Female: 68%, Male: 30%, Non-binary: 2%	Female: 66%, Male: 32%, Non-binary: 2%	0.914
Ethnicity	White: 54%, Asian: 18%, Mixed: 10%, Black: 10%, Other: 8%	White: 53%, Asian: 20%, Mixed: 9%, Black: 12%, Other: 6%	0.931
Medication status	On medication: 56%, No medication: 44%	On medication: 55%, No medication: 45%	0.885
Baseline PHQ-9	Mean: 15.2 (±4.8)	Mean: 15.0 (±5.0)	0.812
Baseline IDS	Mean: 36.7 (±9.4)	Mean: 37.1 (±9.8)	0.847

Table A: Baseline characteristics of completers vs. non-completers. Values are presented as means (± SD) or percentages. p-values are derived from independent t-tests (continuous variables) or χ^2 tests (categorical variables). PHQ-9 and IDS scores are reported only for participants who completed at least the baseline assessment, as these measures were not collected during sign-up. Demographic information is provided for all individuals that passed inclsuion criteria and, thus, were assigned to either the active or control group.

4.2 NHS Talking Therapies and Behavioural Activation Delivery

NHS Talking Therapies (formerly Improving Access to Psychological Therapies, IAPT) is a UK-wide service providing evidence-based, standardised, and manualised psychological treatments for adults with common mental health problems [1, 2]. Within this stepped-care framework, Step 2 interventions for depression consist of low-intensity treatments delivered over 3-6 weekly sessions, typically lasting around one hour each. In the present study, Step 2 comprised manualised behavioural activation following the Health, & Britain) [3] guideline for the treatment and management of depression in adults.

Treatment was delivered by accredited Psychological Wellbeing Practitioners, who receive formal training and regular supervision to ensure fidelity to the intervention protocol. This Step 2 BA format has been evaluated in large-scale randomised controlled trials (e.g., [4]), and is recommended in UK clinical practice for its scalability and cost-effectiveness.

The BA programme focuses on increasing engagement in positively reinforcing activities and reducing avoidance behaviours. Core components in the first three weeks include activity monitoring, activity scheduling, and values-based goal setting. In principle, Step 2 BA may be followed by up to three sessions of cognitive restructuring, though this is rarely implemented in practice.

4.3 Model performance

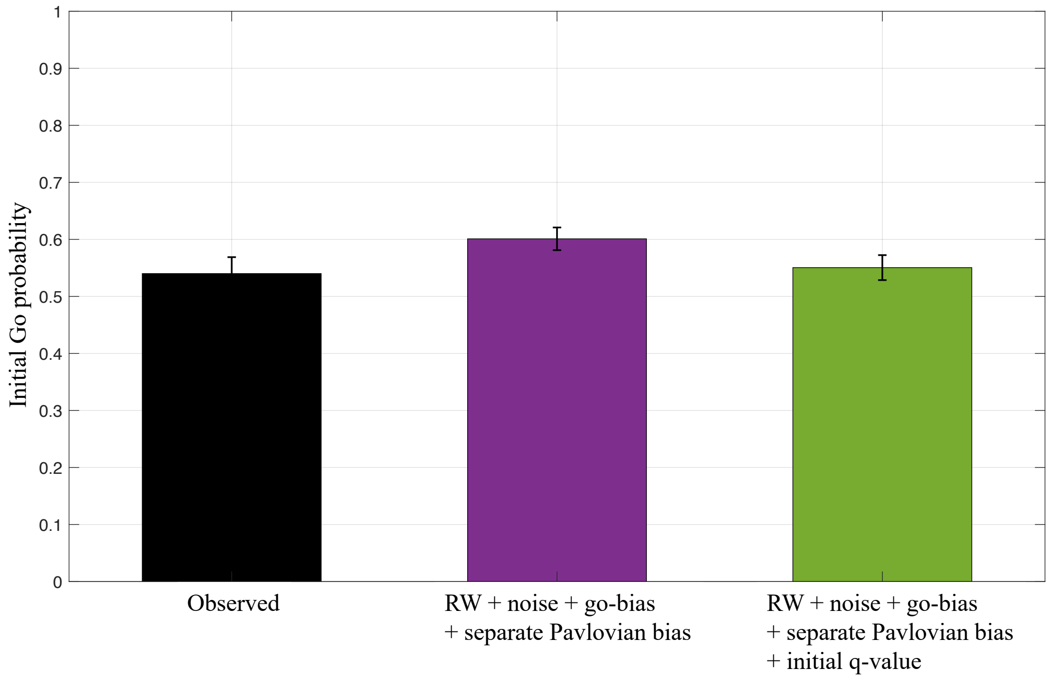

Fig B: ***Contribution of the initial Q-value parameter to model performance.*** Bars show the empirical initial Go probability (black), calculated from the first three trials of each session, compared with predictions from two models. The second-best performing model (purple) corresponds to the winning model without the initial Q-value parameter, whereas the winning model (green) includes this parameter. Error bars indicate variability across participants (± SEM). The model without an initial Q-value parameter overestimates the early Go response observed in the data, whereas the winning model more accurately reproduces this initial response tendency.

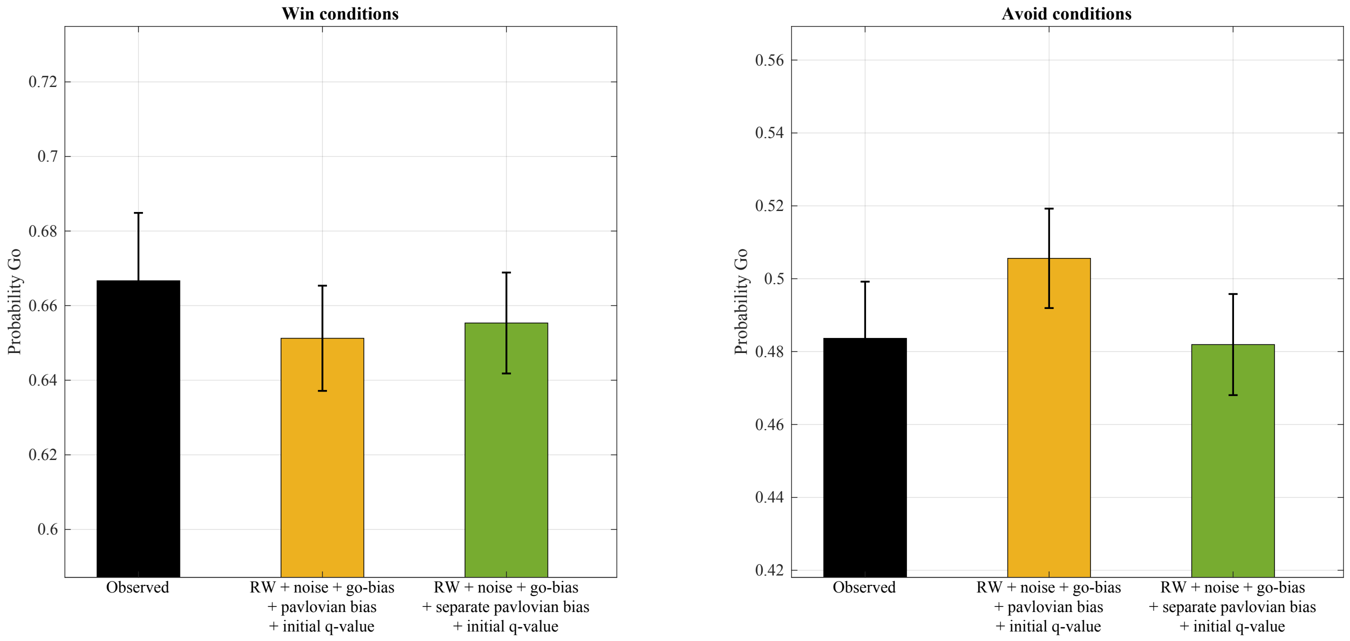

Fig C: ***Probability of Go responses in Win and Avoid conditions for the observed data and model predictions.*** The model with a single Pavlovian bias parameter (yellow) cannot simultaneously capture the higher Go probability in Win conditions and the lower Go probability in Avoid conditions. In contrast, the model with separate Pavlovian bias parameters for Win and Avoid conditions (green) provides a closer match to the empirical data. Both models include the initial Q-value parameter, consistent with the winning model. Error bars indicate variability across participants (± SEM).

4.4 Symptom changes from baseline to assessment 1

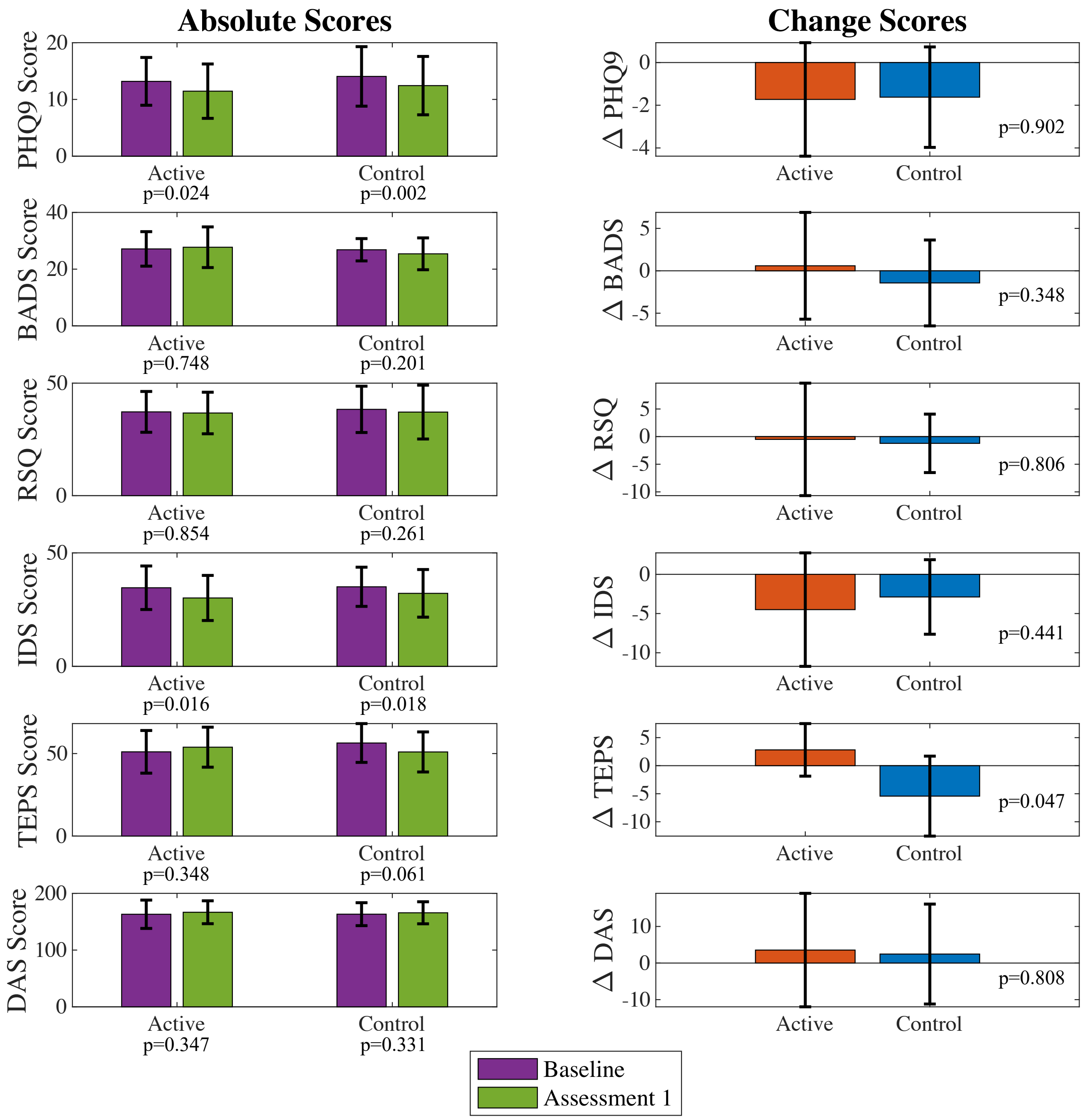

Fig D: ***Questionnaire score summaries for Active and Control groups.*** The figure displays data for six questionnaires: PHQ-9, BADS, RSQ, IDS, TEPS, and DAS. **Left column:** Mean absolute scores (± SD) at Baseline (purple) and Assessment 1 (green) for each group. **Right column:** Mean change scores (Δ = Assessment 1 − Baseline; ± SD) for each group (Active: orange; Control: blue).

4.5 Changes in Pleasure Experience from Baseline to Follow-Up

We focused our follow-up analysis on the Temporal Experience of Pleasure Scale (TEPS) as it was the only measure that showed significant between-group differences at Assessment 1 (prior to control group therapy). Here, we compared TEPS changes between groups at the 6-week follow-up, after both groups had completed behavioral activation therapy. Between-group comparisons were conducted using independent t-tests, with non-parametric Mann-Whitney U tests used when normality assumptions were violated (assessed using the Lilliefors test).

Both groups showed improved pleasure experience, with no significant difference in the magnitude of change between them ($p = 0.375$, $d = 0.30$). This contrasts with Assessment 1 (prior to control group therapy; Fig. 3A), where the active group's TEPS scores improved ($M = +2.82$, $SD = 4.68$) while the control group's worsened ($M = -5.43$, $SD = 7.13$), a significant difference ($p = 0.047$, $d=0.53$). This earlier disparity was not present post-therapy. These findings support that the early differences in changes in pleasure experience between groups were likely related to behavioral activation therapy, as the control group showed similar improvements after receiving the same intervention.

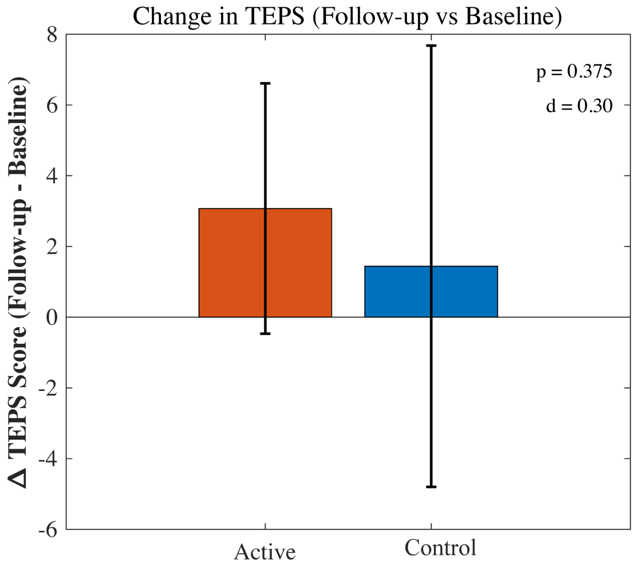

Fig E: ***Change in TEPS (Temporal Experience of Pleasure Scale) scores from baseline to 6-week post-therapy follow-up.*** This chart displays the mean change in TEPS scores from baseline to the 6-week follow-up, after both active (orange) and control (blue) groups completed behavioral activation therapy.

4.6 Go-nogo task behaviour

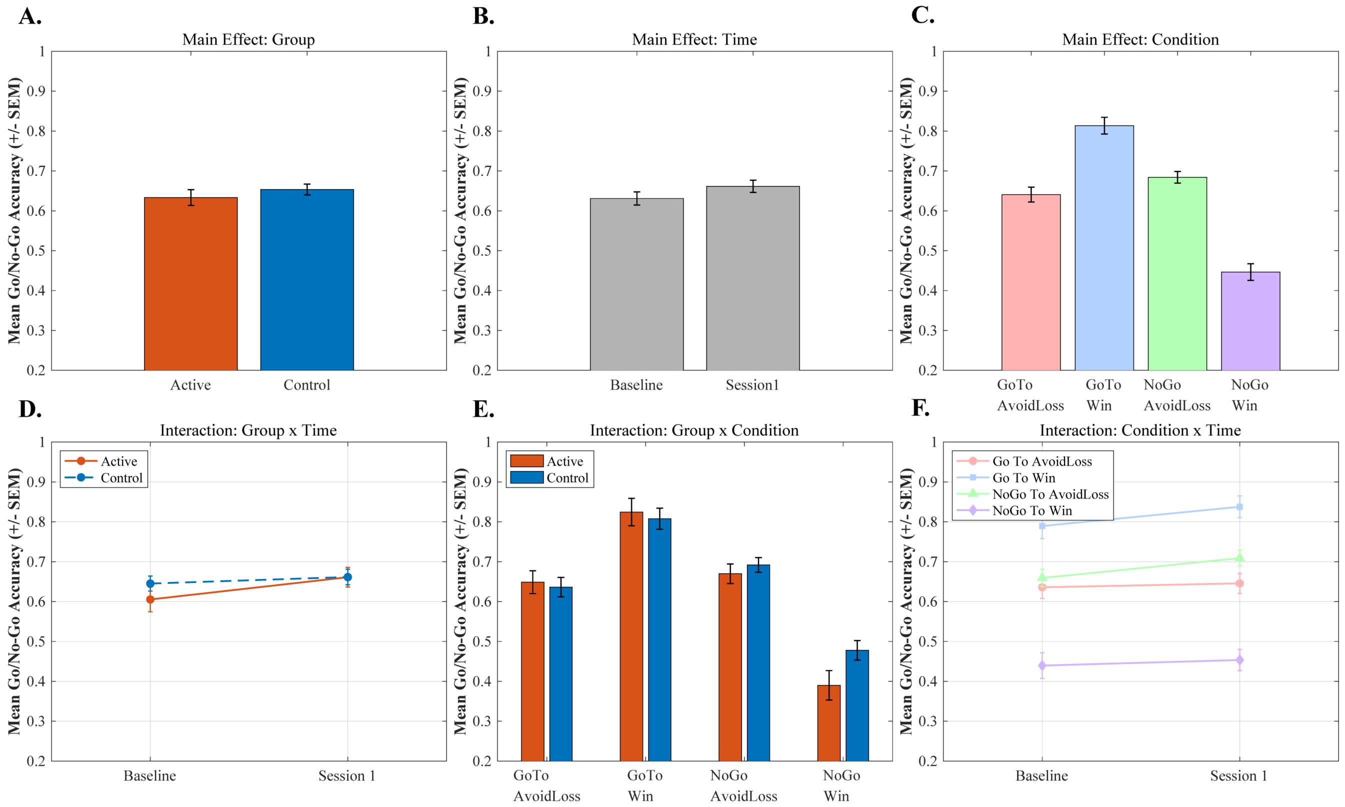

Fig F: ***Main and interaction effects from the Linear Mixed-Effects model on Go/No-Go task accuracy, complementing analyses presented in the main body.*** Mean accuracy (± SEM) is shown. **A.** Main effect of Group (Active vs. Control; $t(456) = -0.24$, $p = 0.807$). **B.** Main effect of Time (Baseline vs. Session 1; $t(456) = 0.14$, $p = 0.892$). **C.** Main effect of Condition (e.g., GoToWin vs. GoToAvoidLoss [reference]: $t(456) = 2.27$, $p = 0.024$; NoGoToAvoidLoss vs. reference: $t(456) \approx 0.00$, $p = 1.0$; NoGoToWin vs. reference: $t(456) = -4.78$, $p < 0.001$). **D.** Group × Time interaction ($t(456) = 0.03$, $p = 0.979$). **E.** Group × Condition interaction (e.g., Control vs. Active on NoGoToWin: $t(456) = 2.04$, $p = 0.061$). **F.** Condition × Time interaction (e.g., GoToWin × Session 1: $t(456) = 0.77$, $p = 0.444$). Lines or bars are colored by group (Active: orange; Control: blue) or condition (GoToAvoidLoss: light red; GoToWin: light blue; NoGoToAvoidLoss: light green; NoGoToWin: light purple).

To examine task performance dynamics, we conducted a linear mixed-effects analysis of Go/No-Go accuracy. The model included main effects of group (Active vs. Control), time (Baseline vs. Session 1), and condition (GoToWin, GoToAvoidLoss, NoGoToWin, NoGoToAvoidLoss), as well as their interactions. Participant-level random effects were included to account for inter-individual variability in task performance. The GoToAvoidLoss condition served as the reference level for condition comparisons. We also examined potential demographic effects by including age and sex as covariates.

4.7 Behavioural interpretation of Pavlovian bias parameters

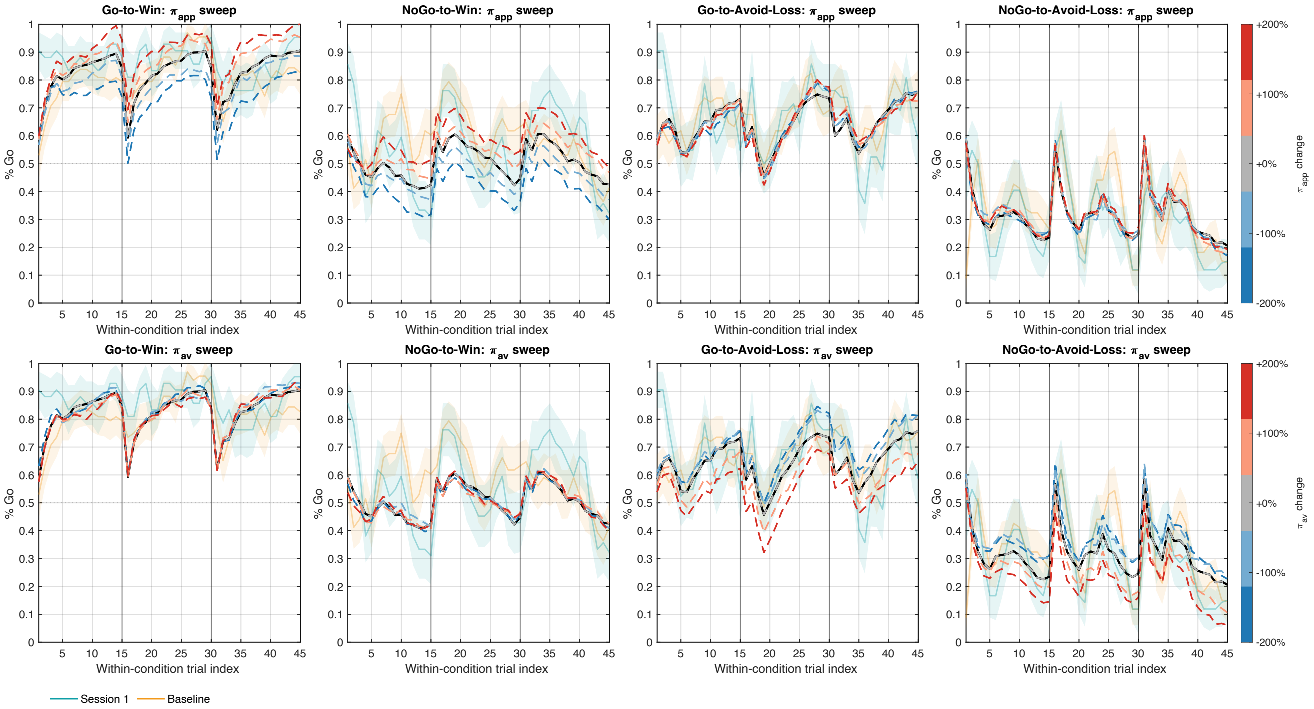

Fig G: Trial-by-trial % Go responses as a function of appetitive and aversive Pavlovian bias parameters in the Active group. Each panel shows the proportion of Go responses (% Go) across within-condition trial indices for each of the four task conditions: Go-to-Win, NoGo-to-Win, Go-to-Avoid-Loss, and NoGo-to-Avoid-Loss. The top row shows predicted trajectories as a function of varying the appetitive Pavlovian bias parameter (relative change from the best-fit estimate for baseline), and the bottom row shows the equivalent sweep for the aversive Pavlovian bias parameter. Solid lines indicate observed % Go at Baseline (orange) and Session 1 (turquoise). Dashed lines indicate model-predicted trajectories: the black dashed line reflects the best-fit model prediction, and coloured dashed lines reflect predictions under parameter sweeps from baseline prediction (red = increase, blue = decrease). Increasing π_{app} increased % Go in both win conditions, while increasing π_{av} reduced % Go in both loss conditions. The observed Baseline-to-Session 1 changes in the Active group were in the direction predicted by the model for both incongruent conditions (NoGo-to-Win: 56% to 58%; Go-to-Avoid-Loss: 63% to 64%), though neither reached significance (NoGo-to-Win: $p = 0.16$; Go-to-Avoid-Loss: $p = 0.37$).

4.8 Task Reliability

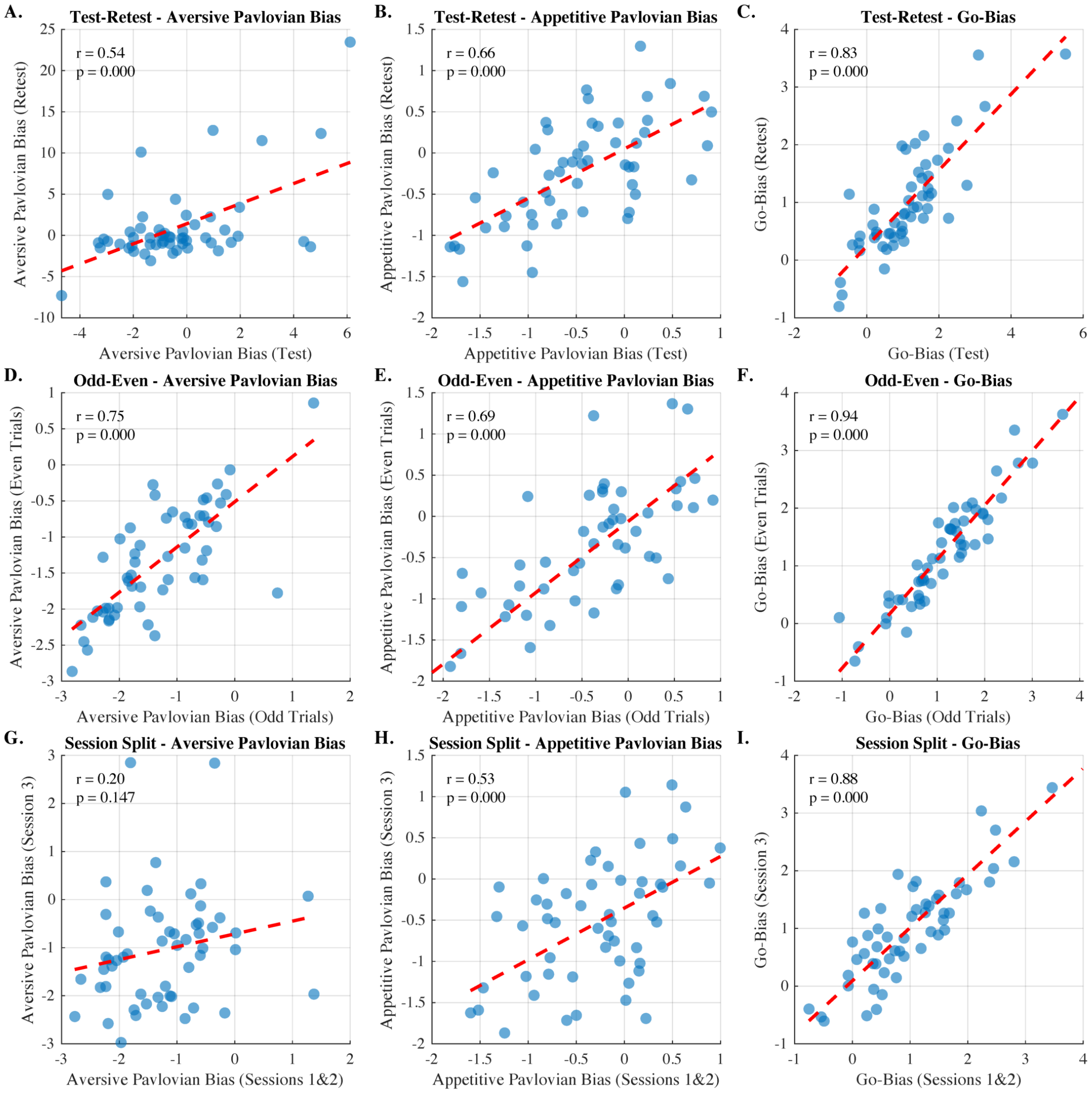

Fig H: ***Reliability Analysis of Model Parameters Across Different Split Methods.*** Correlation analyses of the three key model parameters (Aversive Pavlovian Bias, Appetitive Pavlovian Bias, and Go-Bias) across three different split methods: Test-Retest **(A-C)**, Odd-Even trials **(D-F)**, and Session Split **(G-I)**. Each subplot shows the correlation between parameter estimates from different splits of the data, with Pearson correlation coefficients (r) and corresponding p-values displayed. Red dashed lines indicate the best linear fit. All parameters show significant correlations across splits ($p < 0.001$) except for the Aversive Pavlovian Bias in the Session Split (G, $p = 0.147$). Go-Bias demonstrates the highest reliability across all split methods ($r = 0.83$-0.94), while the Aversive Pavlovian Bias shows more variable reliability ($r = 0.20$-0.75).

Test-retest reliability involved comparing parameter values across multiple assessment timepoints to evaluate the consistency of task parameters over time. Each parameter was assessed individually, with all other parameters held constant, as this approach mirrors how we examine changes in parameters in our main analyses (where we assume only one parameter changes between sessions). This led to Spearman-Brown corrected reliabilities ranging from 0.2 (noise parameter) to 0.8 (go bias) (Fig. HA-C.).

Split-half reliability of the Go/Nogo task was evaluated in two ways: by comparing parameter estimates between the first two rounds combined versus the third round (Fig. HD-F.), and between odd and even trials within sessions (Fig. HG-I.). After applying the Spearman-Brown correction, split-half reliabilities ranged from 0.6 (noise) to 0.9 (go bias), with odd-even reliability similarly ranging from 0.2 (aversive pavlovian bias) to 0.9 (go bias).

Notably, although test-retest reliability for the aversive Pavlovian bias was lower, odd–even reliability was strong ($r = 0.75$), and the test-retest association remained significant after excluding outliers (±3SD, $r = 0.43$, $p = 0.023$), suggesting that reduced longitudinal stability may reflect genuine intervention-related change.

4.9 Parameter Recovery

We generated synthetic data using known parameter values and then fitted our models to this data to assess how well we could recover the true parameters. Parameter recovery analyses confirmed reliable recovery of all parameters with correlations between true and recovered parameters ranging from 0.89 to 0.94.

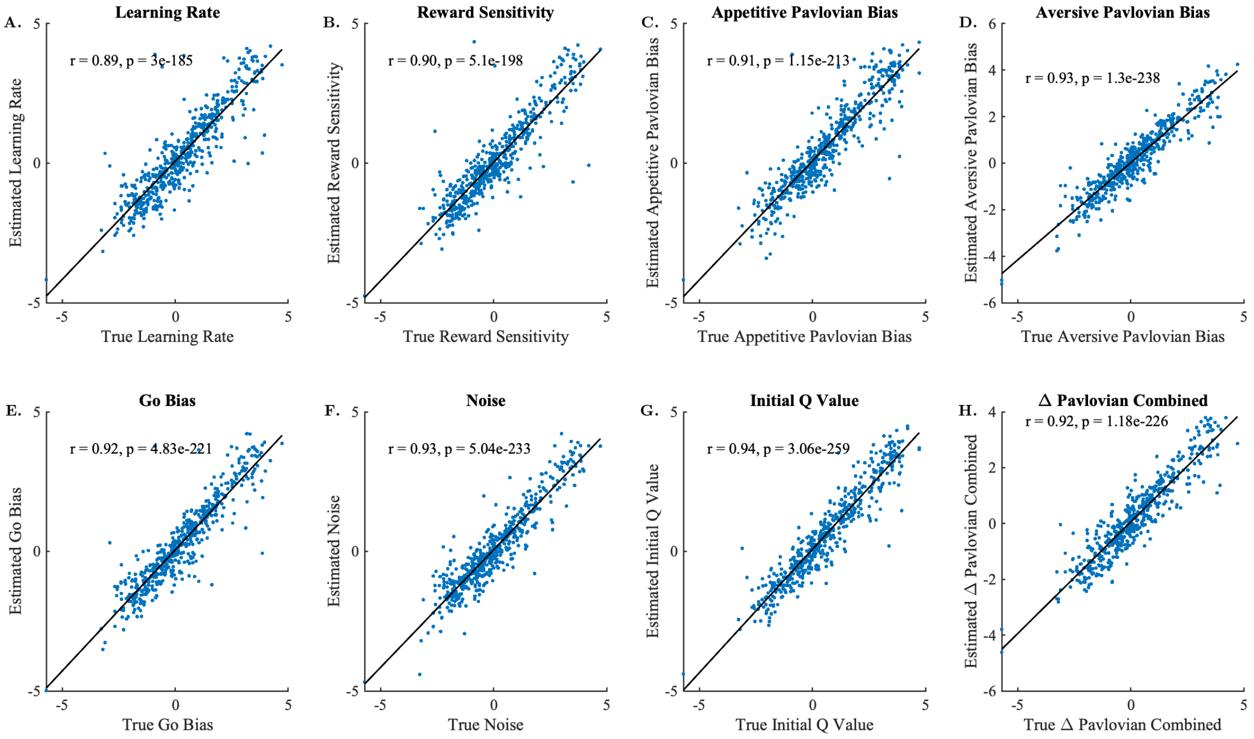

Fig I: ***Parameter Recovery.*** Parameter recovery consistently showed high correlations, with most parameters reaching correlations of 0.9 or above.

4.10 Intention-to-Treat Analyses on Imputed Data

To assess the robustness of our findings, we performed Multiple Imputation by Chained Equations (MICE) for all randomized participants who lacked complete data. We used 20 imputed datasets with 10 iterations per imputation and a convergence threshold of 0.01. The imputation was performed using demographic variables (age, ethnicity, gender, sex, employment) as predictors.

The MICE procedure used chained equations to impute all three outcome variables simultaneously (aversive Pavlovian bias change, combined Pavlovian bias change, TEPS change). In each iteration, each variable was imputed using all other variables (both demographic predictors and other imputed variables) as predictors, allowing the variables to influence each other during the imputation process. Analysis of each completed dataset was performed separately before pooling results using Rubin's rules [5].

We repeated our main analyses on the imputed data. Group differences showed trends in the expected direction but failed to reach significance. For combined Pavlovian bias change, the Active group showed M = 0.067, SD = 0.361 (n = 74) compared to Control group M = -0.156, SD = 0.385 (n = 78), p = 0.059. For TEPS change, the Active group showed M = 0.605, SD = 2.312 (n = 74) compared to Control group M = -0.065, SD = 2.848 (n = 77), p = 0.073.

The mediation analysis revealed that only the b path (Aversive Pavlovian Bias Change → TEPS Change) was significant (b = -1.9307, 95% CI [-3.7967, -0.3622], p = 0.016).

Of the 152 randomized participants, only 60 had both baseline and session 1 data, while 85 had at least baseline data (which were included in the aITT). The remaining participants required imputation. The high proportion of imputed data likely contributed to the loss of treatment signal, explaining why the significant group differences and mediation effects observed in the completer and adjusted ITT analyses were not replicated in the full ITT analysis with MICE imputation.

References

[1] D. M. Clark. (2011). “Implementing NICE guidelines for the psychological treatment of depression and anxiety disorders: the IAPT experience”. *Int Rev Psychiatry*, *23*(4), 318–327.

[2] D. M. Clark. (2018). “Realizing the mass public benefit of evidence-based psychological therapies: the IAPT program”. *Annual review of clinical psychology*, *14*(1), 159–183.

[3] N. I. for Health, & C. E. (Britain). (2022). *Depression in adults: treatment and management*. National Institute for Health and Care Excellence (NICE).

[4] D. A. Richards, D. Ekers, D. McMillan, R. S. Taylor, S. Byford, F. C. Warren, B. Barrett, P. A. Farrand, S. Gilbody, W. Kuyken, et al. (2016). “Cost and Outcome of Behavioural Activation versus Cognitive Behavioural Therapy for Depression (COBRA): a randomised, controlled, non-inferiority trial”. *The Lancet*, *388*(10047), 871–880.

[5] D. B. Rubin. (1978). “Multiple imputations in sample surveys-a phenomenological Bayesian approach to nonresponse”. In: “Proceedings of the survey research methods section of the American Statistical Association”, 20–34.
