# Supplementary material for: Early reduction in aversive Pavlovian bias as a mediator of anhedonia improvement during Behavioural Activation in realistic treatment settings
Source: PLoS Comput Biol. 2026 Jul 14;22(7):e1014439. doi: 10.1371/journal.pcbi.1014439 (PMC13387611; doi:10.1371/journal.pcbi.1014439)
Supplement: Attachment — Submitted filename: Response to Reviewers.pdf [file pcbi.1014439.s002.pdf]

Responses to Reviewers:

We thank the reviewers for their constructive and thorough feedback. We have now revised our manuscript and believe these revisions have made the manuscript substantially stronger. Below we respond (in bold) to each reviewers point (in italics). Changes to the manuscript are made in blue (reviewer 1) and in purple (reviewer 2).

Reviewer #1:

I have previously reviewed this paper for another journal. This version of the manuscript is improved and addresses several of my earlier concerns, though a couple remain unaddressed and are noted below.

We thank the reviewer for taking the time to review our manuscript again and are pleased to hear that this version managed to address most of the earlier concerns. We addressed the remaining issues in the manuscript and respond to each point in bold below (changes to the manuscript are made in blue).

This article examines early changes in performance on an affective go-nogo task, with a focus on Pavlovian biases, and how these changes relate to changes in symptoms in people with depression receiving behavioral activation therapy versus a waitlist control. The findings support a relationship between decreases in anhedonia and reductions in Pavlovian bias. This is an interesting and well-written paper and the results have the potential to be highly impactful. Strengths include the use of a waitlist control and solid parameter recovery and reliability values for task performance.

1) The treatment group did show changes in the TEPS scale measuring anhedonia relative to the waitlist control, but other measures, including measures of depression severity (PHQ-9 and IDS) and behavioral activation-relevant symptoms (BADS) did not show changes. Is this degree of change consistent with the published literature for this treatment modality?

Thank you very much for this question. Figure S4 does in fact show that both PHQ-9 and IDS did show changes, but that there was no difference between the groups at this point. That is, it is not the case that TEPS showed changes where the other broader measures did not but rather that TEPS showed differential changes early on. The question is hence why. One possibility is that the TEPS focuses on momentary hedonic experience, whereas both IDS and PHQ-9 ask participants to reflect on what has been typical over a longer period of time. This may have made the TEPS more sensitive to early differential effects. A second possibility is that early target engagement precedes broader clinical improvement. Anhedonia is generally considered harder to shift than global depressive symptom severity, so if anything, one might have expected differential effects to emerge first on the broader measures. The fact that the opposite pattern was observed (group-specific effects on the harder-to-move outcome but not the easier ones) suggests BA is exerting a specific early influence on hedonic functioning rather than producing a general improvement, consistent with evidence identifying anhedonia as a core target of BA (Webb et al., 2023). Waitlist controls often show no improvement or even worsening, while here they showed an improvement. We note that the 'wait' was actually quite short and this brevity may have been a positive surprise to patients, possibly driving overall positive effects.

We have now added this to the discussion (p. 11 ll. 330-339):
Interestingly, both groups showed reductions in global depressive symptom severity over the early assessment window, whereas only anhedonia showed a group-specific effect. The reasons for the shared improvement in global symptoms might have arisen from the relative brevity of the wait, which may have been a (positive) surprise to participants. The group-specific effect on anhedonia is notable: anhedonia is generally considered harder to shift than global depressive symptom severity, so finding differential effects precisely on this outcome suggests BA is exerting a specific early influence on hedonic functioning. This is consistent with prior work identifying anhedonia as a key target of BA (Webb et al., 2023) and raises the possibility that anhedonia improvements may

precede broader symptomatic change, which is more commonly reported at later treatment endpoints in BA trials (Richards et al., 2016). The focus of the TEPS used to measure anhedonia is more on momentary hedonic experience which may also make it more sensitive to early differential effects than the PHQ-9 and IDS, which rely on retrospective symptom ratings over a longer period.

2) The a priori power analysis was for N=63 per group, but the 3-week assessment had only N=23 in the treatment group. Is it possible that null effects (and possibly significant findings: Loken, E. and Gelman, A. (2017). Measurement error and the replication crisis. Science 355, 584-585. DOI:10.1126/science.aal3618) are due to low power?

We appreciate the reviewer's concern regarding the impact of reduced power on our 3-week findings. We agree that the drop in sample size to N=23 in the treatment group introduces significant uncertainty. To mitigate the risk of a Type-S (significance/direction) error, we performed the robustness analyses mentioned in the manuscript (mITT and full ITT). The fact that the Pavlovian bias and TEPS effects remained significant in the mITT and directionally consistent in the full ITT suggests that the findings are not only artifacts of the smaller completer sample. However, we acknowledge that these analyses do not eliminate the risk of Type-M (magnitude) error (Loken & Gelman, 2017). In a low-power setting, statistically significant effects are likely to be overestimates of the true population effect. We have revised the Limitations to explicitly state that while the direction of our mechanistic findings appears robust, the magnitude of these effects should be interpreted with caution until replicated in a larger sample.

We have now added the following to the limitations (pp.11-12 ll.357-362):
The high attrition rate during treatment limited both our ability to examine longer-term maintenance of effects and the statistical power and precision of early (3-week) analyses. Consequently, null effects on symptom measures should be interpreted as inconclusive rather than evidence of absence, and the magnitude of statistically significant early effects may be overestimated (Type-M error; (Loken & Gelman, 2017)). Most early effects of interest were in the small-to-moderate range, which is difficult to detect reliably with the available sample. Replication in larger samples with improved retention will be necessary to confirm these early mechanistic findings.

We thank the reviewer again for their continued careful and constructive feedback on this work which has made the manuscript scientifically more rigorous.

Reviewer #2:

In the study by Berndt et al., the authors investigate the effect of behavioural activation therapy on Pavlovian biases in an affective go/nogo task as part of the UK NHS talking therapy service. They compare parameters fitted using reinforcement learning between an active and control group. In line with their hypothesis, they find that behavioural activation increases appetitive and reduces aversive Pavlovian biases. They also find that the effect of behavioural activation on anhedonia is mediated by this change in Pavlovian bias. This is a rare gem of a study which applies ideas from computational psychiatry in a realistic treatment setting, bridging between mechanistic theory and patient symptoms. It is a heroic effort and a study that was clearly conducted very carefully. I enjoyed reading it and have a few questions and suggestions below.

We thank the reviewer for their constructive and encouraging comments. We are pleased that the reviewer found the study compelling and valuable in bridging computational psychiatry with routine clinical care. Below, we address each point in turn and describe the changes made to the manuscript (changes in the manuscript are in purple).

1.) Main finding of changes in Pavlovian bias (Fig4): While the study is very well written and the effects seem sound and convincing, explanations and visual illustrations of the data are relatively brief. To get a good grasp of the key effect, some additional analyses and plots would be very helpful and make the manuscript more convincing. For example, plotting the key result in an intuitive way by illustrating the

effect of changing the affective bias in the computational model on some basic behavioural metric. This could maybe be achieved by plotting the tendency to produce a go action (% go) when Vt is <0 or >0, i.e. when the expected value was be positive or negative (for the two groups and two time points).

We thank the reviewer for this helpful suggestion and agree that an intuitive behavioural illustration would strengthen the main findings and make them more accessible for a broader audience. We therefore systematically varied the appetitive and aversive Pavlovian bias parameters in the model and quantified their impact on predicted behaviour. The resulting plots (new Supplementary Fig.S7) show that the two biases have distinct, condition-specific behavioural signatures: increasing appetitive Pavlovian bias selectively increased Go responses in the incongruent reward condition (NoGo-to-Win), whereas increasing aversive Pavlovian bias selectively decreased Go responses in the incongruent punishment condition (Go-to-Avoid-Loss). Congruent conditions showed comparatively minor changes. This figure provides a direct mapping from the two Pavlovian bias parameters to observable behaviour and make the effect in Fig. 4 more transparent.

We added this figure (Fig.S7 now) to the supplements (p.21):

4.7 Behavioural Interpretation of Pavlovian bias parameters

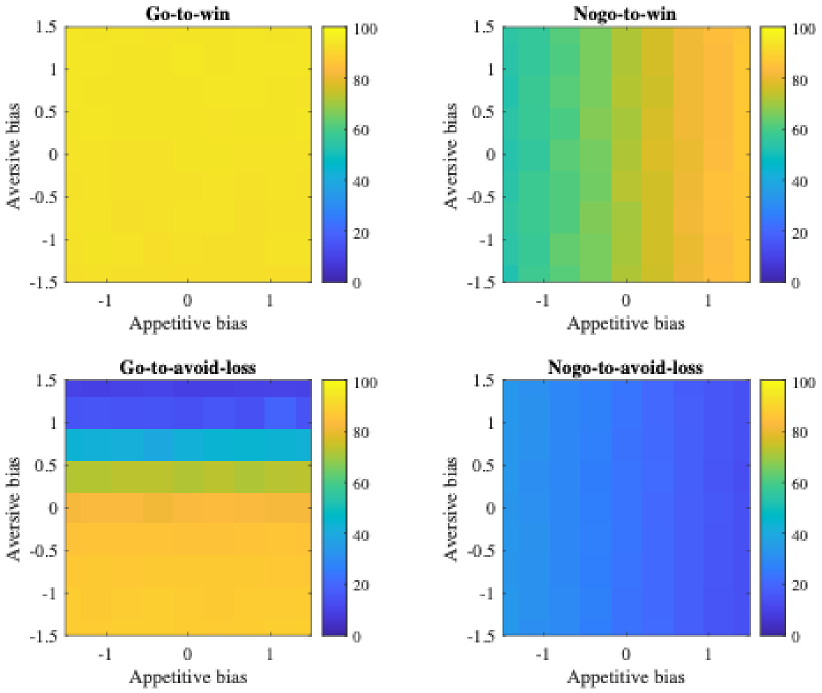

Figure S7: Behavioural consequences of appetitive and aversive Pavlovian bias parameters. Probability of Go responses (%Go; with warmer colours indicating higher probability of go responses and cooler colours lower) simulated from the winning model as a function of appetitive and aversive Pavlovian bias parameters, shown separately for each task condition.

and reference it in our results (p.8 ll.274-276):

To provide an intuitive behavioural interpretation of these parameter changes, we additionally visualised how systematic variation in the appetitive and aversive Pavlovian bias parameters affects the probability of Go responses across task conditions (Supplementary Fig. S7).

2.) Relatedly, it seems that Figure S4E shows a trend-wise interaction driven by an effect in NoGo to win. It seems after behavioural activation, the active group is less accurate at nogo to win. Is this driven by the

same effect captured by the computational modelling analyses, i.e. by expectation of reward making them go (when in this condition, they should not go), or is it due to some other mechanism?

The interpretation suggested (i.e., reward expectation increasing Go responding and thus impairing NoGoToWin performance) is exactly what the modelling framework would predict. As shown in Supplementary Figure S4E (now Figure S6E), NoGoToWin accuracy is numerically lower in the Active group than in the control group, which is directionally consistent with what the modelling would predict for increased appetitive Pavlovian bias in this incongruent condition. However, the corresponding longitudinal Time×Group×Condition interaction in raw accuracy (Figure 3C) did not reach statistical significance (p.7 ll. 254-255). We therefore refrain from interpreting the pattern in the supplementary figure as a reliable BA-related behavioural change or attributing it to a specific mechanism based on accuracy alone.

3.) Model fits (Fig4): While some models are visually clearly better than others in Figure 4B when overlaid on the data, even the winning model does not capture all trends and variations. Because there are no error bars around the actual data line (black line in Fig 4B), it is a bit difficult to see how consistent some of the deviations between model and data are across participants. Error bars, some more information on individual goodness of fit, and on what aspects of the data can consistently (?) not be explained well and why would help assess the goodness of the winning model. Also, some more descriptive statistics, such as the percentage of choices correctly explained (across groups, time points, and task conditions).

This is an important point. We agree that, when overlaid in Fig. 4B, the top three models are visually difficult to distinguish because their predictions lie very close together, and that the absence of uncertainty around the empirical estimates makes it harder to judge how systematic the remaining deviations are across participants. To address this, we have added two supplementary figures (Fig. S2, p.18 & Fig. S3, p.18) that provide a clearer assessment of goodness-of-fit for the winning model.

First, we examined the role of the initial Q-value parameter, which allows the model to capture participants' initial action tendencies before learning occurs. In the model comparison reported in the manuscript, the second-best performing model was identical to the winning model except that it did not include the initial Q-value parameter. To quantify early response tendencies, we calculated the empirical initial Go probability using the first three trials of each session. As shown in now Supplementary Fig. S2, the model without an initial Q-value parameter systematically overestimates this early Go bias, whereas the winning model more accurately reproduces the observed initial Go probability.

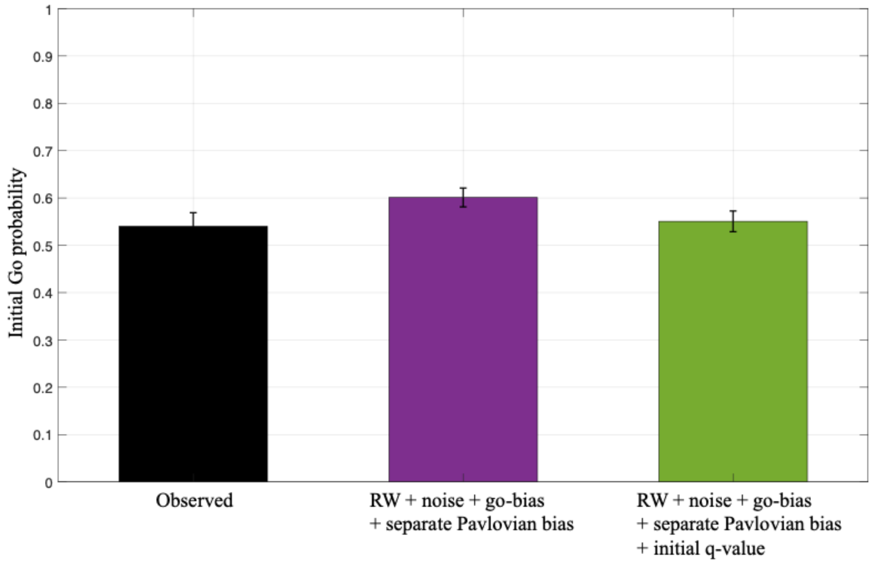

Figure S2: Contribution of the initial Q-value parameter to model performance. Bars show the empirical initial Go probability (black), calculated from the first three trials of each session, compared with predictions from two models. The second-best performing model (purple) corresponds to the winning model without the initial Q-value parameter, whereas the winning model (green) includes this parameter. Error bars indicate variability across participants (± SEM). The model without an initial Q-value parameter overestimates the early Go response observed in the data, whereas the winning model more accurately reproduces this initial response tendency.

Second, we examined whether a single Pavlovian bias parameter is sufficient to account for behaviour, or whether separate Pavlovian biases for Win and Avoid conditions are required. In the model comparison reported in the manuscript, the third-best performing model included a single Pavlovian bias parameter, whereas the winning model included separate Pavlovian biases. Because the winning model also included an initial Q-value parameter, we additionally evaluated a model that combined a single Pavlovian bias with the initial Q-value parameter to ensure that the advantage of separate Pavlovian biases was not driven by the inclusion of the initial Q-value term. As shown in Supplementary Fig. S3, the model with a single Pavlovian bias cannot simultaneously capture the higher Go probability in Win conditions and the lower Go probability in Avoid conditions, whereas the model with separate Pavlovian bias parameters provides a closer match to the empirical data across conditions.

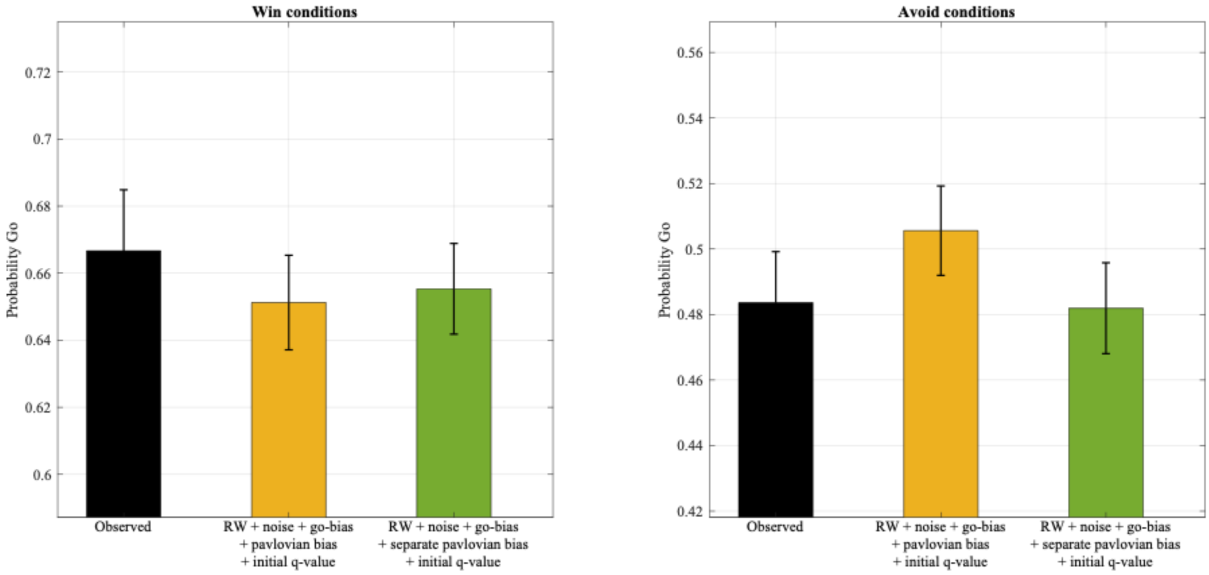

Figure S3. Probability of Go responses in Win and Avoid conditions for the observed data and model predictions. The model with a single Pavlovian bias parameter (yellow) cannot simultaneously capture the higher Go probability in Win conditions and the lower Go probability in Avoid conditions. In contrast, the model with separate Pavlovian bias parameters for Win and Avoid conditions (green) provides a closer match to the empirical data. Both models include the initial Q-value parameter, consistent with the winning model. Error bars indicate variability across participants (± SEM).

and reference it in our results (p.7 ll.260-262):

To further illustrate the contribution of specific model components, Supplementary Figs. S2 and S3 show how the inclusion of the initial Q-value parameter improves the model's ability to capture early response tendencies, and how separate Pavlovian biases for Win and Avoid conditions better reproduce the observed differences in Go responding across conditions.

4.) Behavioural activation therapy (Fig S2): For someone less familiar with the precise expected effects of

behavioural activation at the time scale studied, can the behavioural activation therapy be seen as having been successful/effective if symptom changes are similar in the active and control groups across all measures but one (TEPS)? Is this expected? It would be helpful to comment and expand on this in the introduction or discussion.

Thanks for raising this important observation. Figure S4 does in fact show that both PHQ-9 and IDS did show changes, but that there was no difference between the groups at this point. That is, it is not the case that TEPS showed changes where the other broader measures did not but rather that TEPS showed differential changes early on. The question is hence why. One possibility is that the TEPS focuses on momentary hedonic experience, whereas both IDS and PHQ-9 ask participants to reflect on what has been typical over a longer period of time. This may have made the TEPS more sensitive to early differential effects. A second possibility is that early target engagement precedes broader clinical improvement. Anhedonia is generally considered harder to shift than global depressive symptom severity, so if anything, one might have expected differential effects to emerge first on the broader measures. The fact that the opposite pattern was observed (group-specific effects on the harder-to-move outcome but not the easier ones) suggests BA is exerting a specific early influence on hedonic functioning rather than producing a general improvement, consistent with evidence identifying anhedonia as a core target of BA (Webb et al., 2023). Waitlist controls often show no improvement or even worsening, while here they showed an improvement. We note that the 'wait' was actually quite short and this brevity may have been a positive surprise to patients, possibly driving overall positive effects.

We, thus, added to the discussion (p. 11 ll.330-338):
Interestingly, both groups showed reductions in global depressive symptom severity over the early assessment window, whereas only anhedonia showed a group-specific effect. The reasons for the shared improvement in global symptoms might have arisen from the relative brevity of the wait, which may have been a (positive) surprise to participants. The group-specific effect on anhedonia is notable: anhedonia is generally considered harder to shift than global depressive symptom severity, so finding differential effects precisely on this outcome suggests BA is exerting a specific early influence on hedonic functioning. This is consistent with prior work identifying anhedonia as a key target of BA (Webb et al., 2023) and raises the possibility that anhedonia improvements may precede broader symptomatic change, which is more commonly reported at later treatment endpoints in BA trials (Richards et al., 2016). The focus of the TEPS used to measure anhedonia is more on momentary hedonic experience which may also make it more sensitive to early differential effects than the PHQ-9 and IDS, which rely on retrospective symptom ratings over a longer period.

5.) Figure 5B, relationship TEPS and aversive bias: Was there a difference between groups? It seems that the correlation might be driven more by the control group and thus a change in TEPS not caused by behavioural activation? What are the authors' thoughts on this? And how does this relate to the mediation analysis?

The reviewer raises an extremely relevant point. Our original regression included group as a covariate (p=0.037), but we did not explicitly test the group*aversive bias interaction. We have now done so: there was a trend, but the interaction did not reach significance (p=0.063), while aversive Pavlovian bias remained significant (p=0.041). Both findings are reassuring. The retained significance of aversive bias confirms that the relationship with anhedonia is robust, while the (trend) non-significant interaction suggests it holds across both groups rather than being driven by one alone. Although we do acknowledge some arbitrariness regarding the p=0.05 threshold, we think that this interpretation is overall consistent with the other results. Importantly, it aligns with the mediation analysis, which specifically tests whether changes in aversive bias account for BA's effect on anhedonia.

We now explicitly mention this in the result section (p.8 ll.282-283):
This association remained significant (p=0.041) after including a group*aversive Pavlovian bias interaction (p=0.063), suggesting the relationship was not specific to either group.

6.) Reliability (Fig S5): It is great to see reliability analyses being conducted with so much care. The reliability of the aversive Pavlovian bias looks poorer than other parameters: this might be an illusion due to the plot axes not being symmetrical in Fig S5 which should be corrected but could also potentially be because only a handful of datapoints are driving the positive correlation for this parameter. Have the authors got any thoughts on this or looked into it further? Have they considered a robust regression analysis less driven by outliers? It is one of the key parameters for the paper, so its reliability seems important. I am aware that many studies still do not report test-retest reliability and that the reliability is overall good and I commend the authors for looking at this so carefully.

We thank the reviewer for highlighting the importance of reliability for this key parameter and for raising the possibility that the observed test-retest correlation might be driven by a small number of datapoints. We repeated the test-retest analysis for the aversive Pavlovian bias after excluding outliers (± 3SD). The association remained statistically significant, although reduced (r=0.43, p=0.023). Importantly, the aversive Pavlovian bias demonstrated strong odd-even reliability (r=0.75), comparable to other parameters. This indicates good internal consistency of the parameter estimation within session. The lower test-retest reliability should also be interpreted in light of the study design: our hypothesis and empirical findings indicate that behavioural activation systematically modulated this parameter (decrease in the active group, increase in controls). Such intervention-related change necessarily reduces rank-order stability across sessions.

We have now added this explanation to the supplements (pp.22-23 ll.569-571):
Notably, although test-retest reliability for the aversive Pavlovian bias was lower, odd-even reliability was strong (r = 0.75), and the test-retest association remained significant after excluding outliers (±3SD, r = 0.43, p = 0.023), suggesting that reduced longitudinal stability may reflect genuine intervention-related change.

Minor
1.) How is the combined Pavlovian bias defined?

The combined Pavlovian bias is a separate free parameter estimated during model fitting. We fit both sessions jointly. To model session-related changes, we introduced a single additional parameter that captures a coupled shift in Pavlovian bias between sessions. Specifically, this parameter induces a simultaneous increase in appetitive Pavlovian bias and decrease in aversive Pavlovian bias, rather than allowing two independent changes.

We now make this more explicit in the method section (p.5 ll.197-202):
We constructed separate change models in which each parameter was allowed to vary between sessions while all other parameters were held constant. Thus, for each parameter, a corresponding change model estimated a baseline value and a session-related shift.
For the Pavlovian bias change model, the session shift was implemented as a single additional parameter inducing a coupled change in appetitive and aversive Pavlovian bias. Specifically, this parameter increased the appetitive bias while decreasing the aversive bias by the same magnitude, thereby enforcing opposing changes rather than allowing them to vary independently.

2.) The introduction could be more specific about the precise RL deficits seen in depression, it is kept quite vague.

We thank the reviewer for this suggestion. Depression is a highly heterogeneous condition and RL findings vary considerably across studies and patient subgroups, making it difficult to make specific claims about RL deficits in depression as a whole without overstating the evidence. We

have therefore chosen to keep the introduction focused on the Pavlovian processes specifically relevant to BA. BA at its core has an exposure element, where individuals are asked to act against their absent positive drive and against their present negative drive and changes in Pavlovian bias make considerable sense as a specific mechanistic target in this context. We felt this more focused framing was better supported by the existing literature than a broader characterisation of RL deficits in depression.

3.) If all findings were hypothesised a priori, did the authors consider preregistering the study? If they did, please cite the repository.

This study was not pre-registered. We do agree, however, that this is better practice and we should do this in the future.

4.) Given the 130 minutes per time point could be completed within a 7-day window, more detail on actual completion times/spread should be added to the supplement, if possible.

We agree that additional detail on within-week completion would be ideal. However, we cannot meaningfully report spread relative to the intended 7-day window because the window was anchored to each participant's weekly therapy session, and the timing of those sessions varied across participants. As a result, raw completion dates across the calendar week are not comparable and would primarily reflect differences in when therapy occurred, rather than task-burden or adherence. What we can add is a proxy for whether completion was concentrated vs distributed: 73% of participants completed all tasks on the same calendar day, suggesting that most did not spread the battery across multiple days (although same-day completion does not necessarily imply a single uninterrupted sitting). Total completion time averaged 97 min (SD = 16).

We have added this to the supplements (p.17 ll.521-522):
Across participant/timepoints, mean total completion time was 97 min (SD = 16), and 73% completed all tasks on the same calendar day.

5.) The PHQ-9 asks about suicidal thoughts – were any procedures in place to offer additional support if a patient reported suicidal ideation?

Yes. The PHQ-9 is routinely administered as part of NHS Talking Therapies, and this study was embedded within this service, building directly on its well-established structures for monitoring and responding to suicide risk. A safeguarding protocol was in place whereby any endorsement of suicidal ideation would have been immediately flagged to the study's clinical lead (psychiatrist) for appropriate risk management and referral to support services.

We have added this to the methods (p.3 ll.137-139):
The study was conducted within NHS Talking Therapies, where PHQ-9 administration is routine practice. A safeguarding protocol was in place. Endorsement of suicidal ideation triggered review by the study's clinical lead (psychiatrist) and signposting or referral to appropriate support services.

6.) Figure1: the colours in C are not clear/explained and boxes not aligned.

We have now clarified these in the figure caption (p.3).

A. The task conditions consisted of four combinations of response type (Go/NoGo) and outcome valence (Win/Avoid Loss), forming the four experimental conditions. In this figure, colors are used to represent these different conditions and indicate the corresponding stimulus types; in the actual

task, participants saw distinct stimuli (e.g., boat, bike, etc.) - not colors - for each condition. B. Trial structure and feedback system displaying point outcomes (0, 1, -1) for Go (button press) and NoGo (no button press) responses. Correct actions in win conditions yielded rewards, while correct actions in avoid-loss conditions prevented losses. C. Task structure included three 60-trial blocks, totaling 180 trials per session. Different colors between trial-blocks represent four different stimulus types used in this round.

7.) A few more sentences in the discussion expanding on how Pavlovian processes might be modified in depression and made a potential treatment target in the future would be helpful.

We have now added the following to the discussion (p.11 ll.343-347):
It also raises the possibility that BA may work in part by directly targeting the automatic aversive avoidance and blunted appetitive motivation that characterise Pavlovian biases in depression, encouraging approach behaviour in contexts where avoidance has become the default, and in doing so restoring more normal patterns of hedonic engagement. This would suggest that individuals with stronger aversive Pavlovian bias at baseline may benefit most from BA, motivating future work to test this as a predictor of treatment response and a marker for treatment stratification.

8.) I suggest adding the computational model specifications to the main part of the manuscript, instead of the supplement.

We have moved this to the main part. (pp.4-5 ll.174-193)

We thank the reviewer for their thoughtful feedback, which has helped us improve the clarity of the figures, the evaluation of model adequacy, and the framing of the findings.
