# Supplementary material for: Early reduction in aversive Pavlovian bias as a mediator of anhedonia improvement during Behavioural Activation in realistic treatment settings
Source: PLoS Comput Biol. 2026 Jul 14;22(7):e1014439. doi: 10.1371/journal.pcbi.1014439 (PMC13387611; doi:10.1371/journal.pcbi.1014439)
Supplement: Attachment — Submitted filename: PLOSCB_resp_v3.pdf [file pcbi.1014439.s003.pdf]

Responses to Reviewer:

Below we respond (in bold) to each point (in italics). Changes to the manuscript are made in green.

Reviewer #2:

Thank you the work you put into revising the manuscript, which I believe helped increase the clarity and robustness of what was already a very interesting report. I have only one small comment left:

In response to my first point, the authors added Figure S7 which is a theoretical depiction of how different parameters in the model map onto different behaviours. This is helpful and would predict strong effects on Nogo-to-win and Go-to-avoid-loss trials. Apologies if my initial comment was confusing, but I was actually asking for the actual data to be plotted in an intuitive way so that readers could understand the change in the combined model bias parameters induced by BA therapy in a way that is intuitive and easy to follow. Maybe this is not possible, and of course the raw data leading to these results are shown in Figure 3C, but it takes a while for the reader to link the computational effect shown in Fig 5A to the raw data in 3C and to their key message. This could be made easier for the reader and thus potentially mean more people immediately understand what the authors are trying to convey, making the manuscript more accessible. For example, if indeed the appetitive bias has increased and the aversive bias has diminished, then the prediction would be that the largest effects are on the purple and green Nogo-to-win and Go-to-avoid-loss conditions (in active vs control). It seems BA-mediated effects are particularly pronounced (visually) in the no-go-to-win. Is it fair to conclude then that the effect on the combined parameter Δ combined is more/mostly/predominantly driven by NoGo to win and reflects an increased appetitive Pavlovian bias more than a reduced aversive bias? I acknowledge that (in response to my point 2) the authors refrain from interpreting this effect (which is similarly reflected in Fig S6E) because it is not significant in a triple interaction of Time x Group x Condition. However, it seems if the model predicts changes particularly in NoGo to Avoid Loss or NoGo to Win, and the combined bias change is significant, a post-hoc test focusing on these two conditions would be justified to fully portray and explain the aspects of the data that drive the model-derived combined bias effect. I think this would be an interesting addition as the reader is left wondering whether the Pavlovian bias change was similar in the appetitive and aversive domains which does not seem to be the case (and I wasn't sure if it squares well with this statement in the discussion "This would suggest that individuals with stronger aversive Pavlovian bias at baseline may benefit most from BA".). Thanks for considering this. I would leave it up to the authors to consider making this change.

We thank the reviewer for this important clarification. We have now attempted to address this properly.

The original behavioural analysis was conducted using linear mixed-effects models on all participants with at least baseline data (mITT), which is why the condition-level trajectories in the Figure 3C do not straightforwardly reflect the model-predicted pattern. We realised that our original wording suggested that all anaylsis were done on the completer dataset (which is true for all other analysis) and have now clarified this directly in the method section (p.4 l.173):

All analyses were initially conducted on a complete-case (per-protocol), unless explicitly stated otherwise.

And p.4 ll.183-184
To analyze task performance dynamics over time, we modeled overall accuracy using a mixed-effects model (supplementary materials section 4.6). The linear mixed-effects model was initially conducted on all participants with at least baseline data (mITT).

And p.5 ll.236-239

To directly link the model-derived $\Delta\pi$combined effect to observable behaviour, post-hoc Wilcoxon signed-rank tests were conducted in Active group completers (n=23), comparing % Go responses

between Baseline and Session 1 for the two incongruent conditions (NoGo-to-Win and Go-to-Avoid-Loss), where Pavlovian bias competes with the correct instrumental response (see Supplementary Figure S7 and Section 4.7).

We replaced Figure S7 with a new figure (Figure S7, p.21). We also wish to apologise for the original Figure S7 submitted in the previous revision, which inadvertently used an incorrect version of the model in which parameter effects of the Pavlovian Biases were restricted to the incongruent conditions by construction (this is not the model version we used in the original manuscript). While the predicted directions of effects were the same for the incongruent conditions, it did not show the effects of these Biases on the congruent ones. We are very sorry about this oversight. The new figure uses the full model and shows trial-by-trial % Go responses across all four task conditions, with parameter sweeps for both π_app (top row) and π_av (bottom row). Solid lines indicate observed group mean % Go at Baseline (orange) and Session 1 (turquoise. Dashed lines indicate model-predicted trajectories: the black dashed line reflects the best-fit model prediction for baseline, and coloured dashed lines reflect predictions under parameter sweeps (from baseline), with colour encoding the direction and magnitude of change (red = increase, blue = decrease).

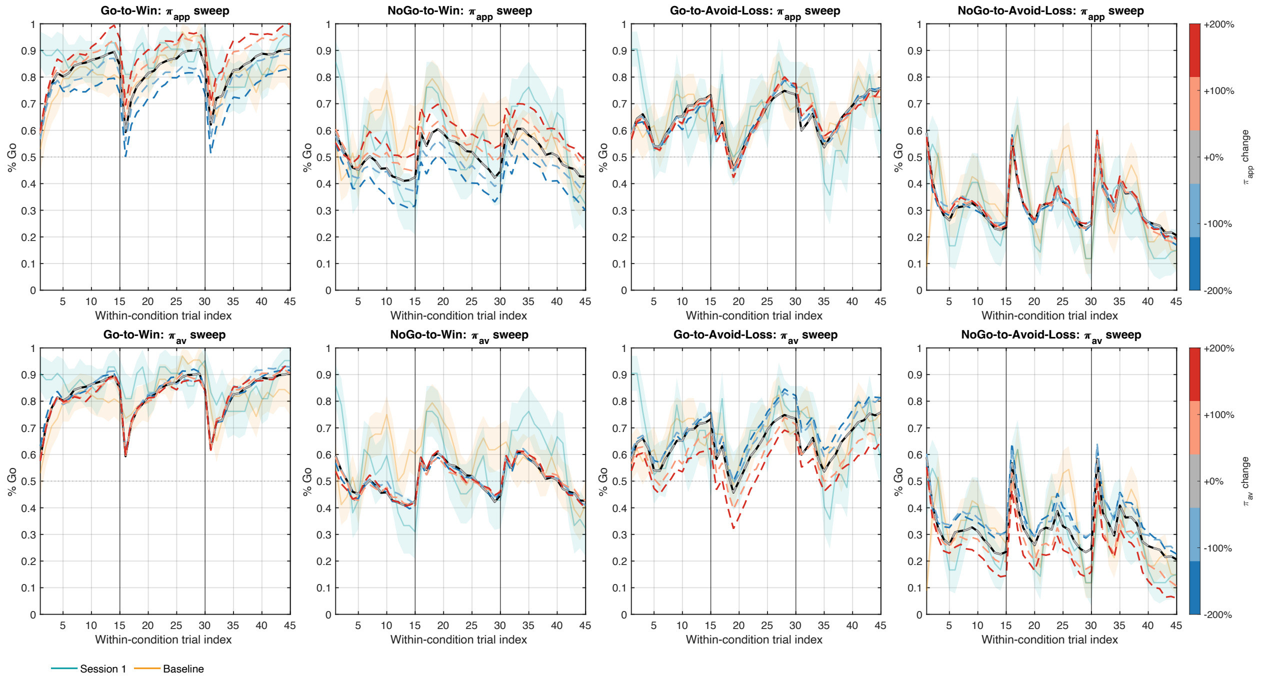

Figure S7. Trial-by-trial % Go responses as a function of appetitive and aversive Pavlovian bias parameters. Each panel shows the proportion of Go responses (% Go) across within-condition trial indices for each of the four task conditions: Go-to-Win, NoGo-to-Win, Go-to-Avoid-Loss, and NoGo-to-Avoid-Loss. The top row shows predicted trajectories as a function of varying the appetitive Pavlovian bias parameter (relative change from the best-fit estimate for baseline), and the bottom row shows the equivalent sweep for the aversive Pavlovian bias parameter. Solid lines indicate observed % Go at Baseline (orange) and Session 1 (turquoise). Dashed lines indicate model-predicted trajectories: the black dashed line reflects the best-fit model prediction, and coloured dashed lines reflect predictions under parameter sweeps from baseline prediction (red = increase, blue = decrease). Increasing π_app increased % Go in both win conditions, while increasing π_av reduced % Go in both loss conditions. The observed Baseline-to-Session 1 changes in the Active group were in the direction predicted by the model for both incongruent conditions (NoGo-to-Win: 56% to 58%; Go-to-Avoid-Loss: 63% to 64%), though neither reached significance (NoGo-to-Win: $p = 0.16$; Go-to-Avoid-Loss: $p = 0.37$).

As shown in the new Figure S7, both incongruent conditions show small increases in % Go from Baseline to Session 1 in the Active group (NoGo-to-Win: 56% to 58%; Go-to-Avoid-Loss: 63% to 64%),

which is in the direction predicted by an increase in appetitive Pavlovian bias (NoGo-to-Win) and a reduction in aversive Pavlovian bias (Go-to-Avoid-Loss). However, post-hoc Wilcoxon signed-rank tests conducted in completers did not reach significance for either condition (NoGo-to-Win: $p = 0.16$; Go-to-Avoid-Loss: $p = 0.37$). This is consistent with our earlier decision to refrain from condition-level interpretation given the absence of a significant Time × Group × Condition interaction, and is unsurprising given the limited number of completers in the Active group.

We hope this revised figure now addresses what the reviewer had originally intended, and apologise again for the initial misunderstanding.
