# Supplementary material for: Early reduction in aversive Pavlovian bias as a mediator of anhedonia improvement during Behavioural Activation in realistic treatment settings
Source: PLoS Comput Biol. 2026 Jul 14;22(7):e1014439. doi: 10.1371/journal.pcbi.1014439 (PMC13387611; doi:10.1371/journal.pcbi.1014439)
Supplement: Peer review history

# Peer review: Decision Letter 0

**DOI:** 10.1371/journal.pcbi.1014439.r001

**Authors:** Wolfgang Einhäuser, Marieke Karlijn van Vugt

28 Jan 2026

PCOMPBIOL-D-25-02123

Early reduction in aversive Pavlovian bias as a mediator of anhedonia improvement during Behavioural Activation in realistic treatment settings

PLOS Computational Biology

Dear Dr. Berndt,

Thank you for submitting your manuscript to PLOS Computational Biology. After careful consideration, we feel that it has merit but does not fully meet PLOS Computational Biology's publication criteria as it currently stands. Therefore, we invite you to submit a revised version of the manuscript that addresses the points raised during the review process.

Please submit your revised manuscript by Mar 30 2026 11:59PM. If you will need more time than this to complete your revisions, please reply to this message or contact the journal office at ploscompbiol@plos.org. When you're ready to submit your revision, log on to https://www.editorialmanager.com/pcompbiol/ and select the 'Submissions Needing Revision' folder to locate your manuscript file.

Please include the following items when submitting your revised manuscript:

* A letter that responds to each point raised by the editor and reviewer(s). You should upload this letter as a separate file labeled 'Response to Reviewers'. This file does not need to include responses to formatting updates and technical items listed in the 'Journal Requirements' section below.

* A marked-up copy of your manuscript that highlights changes made to the original version. You should upload this as a separate file labeled 'Revised Manuscript with Track Changes'.

* An unmarked version of your revised paper without tracked changes. You should upload this as a separate file labeled 'Manuscript'.

If you would like to make changes to your financial disclosure, competing interests statement, or data availability statement, please make these updates within the submission form at the time of resubmission. Guidelines for resubmitting your figure files are available below the reviewer comments at the end of this letter

We look forward to receiving your revised manuscript.

Kind regards,

Wolfgang Einhäuser

Academic Editor

PLOS Computational Biology

Marieke van Vugt

Section Editor

PLOS Computational Biology

**Additional Editor Comments:**

As you will see from their reviews, the reviewers are generally positive about your approach, but raise some critical methological issues that need to be addressed carefully befor a final decision can be made.

**Journal Requirements:**

1) Please ensure that the CRediT author contributions listed for every co-author are completed accurately and in full.

At this stage, the following Authors/Authors require contributions: Lioba Chiara Sophie Berndt, Daisy Crawley, Ruslana Tymchyk, Anna Hall, Jakub Onysk, Tore Erdmann, Elliott Wimmer, Isabel Berwian, Agnes Norbury, and Quentin Huys. Please ensure that the full contributions of each author are acknowledged in the "Add/Edit/Remove Authors" section of our submission form.

The list of CRediT author contributions may be found here: https://journals.plos.org/ploscompbiol/s/authorship#loc-author-contributions

2) We ask that a manuscript source file is provided at Revision. Please upload your manuscript file as a .doc, .docx, .rtf or .tex. If you are providing a .tex file, please upload it under the item type u2018LaTeX Source Fileu2019 and leave your .pdf version as the item type u2018Manuscriptu2019.

3) Please provide an Author Summary. This should appear in your manuscript between the Abstract (if applicable) and the Introduction, and should be 150-200 words long. The aim should be to make your findings accessible to a wide audience that includes both scientists and non-scientists. Sample summaries can be found on our website under Submission Guidelines:

https://journals.plos.org/ploscompbiol/s/submission-guidelines#loc-parts-of-a-submission

4) Please upload all main figures as separate Figure files in .tif or .eps format. For more information about how to convert and format your figure files please see our guidelines:

https://journals.plos.org/ploscompbiol/s/figures

5) We notice that your supplementary Figures, and information are included in the manuscript file. Please remove them and upload them with the file type 'Supporting Information'. Please ensure that each Supporting Information file has a legend listed in the manuscript after the references list.

6) Please amend your detailed Financial Disclosure statement. This is published with the article. It must therefore be completed in full sentences and contain the exact wording you wish to be published.

State what role the funders took in the study. If the funders had no role in your study, please state: "The funders had no role in study design, data collection and analysis, decision to publish, or preparation of the manuscript.".

If you did not receive any funding for this study, please simply state: u201cThe authors received no specific funding for this work.u201d

**Reviewers' comments:**

Reviewer's Responses to Questions

**Comments to the Authors:**

**Please note here if the review is uploaded as an attachment.**

Reviewer #1: I have previously reviewed this paper for another journal. This version of the manuscript is improved and addresses several of my earlier concerns, though a couple remain unaddressed and are noted below.

This article examines early changes in performance on an affective go-nogo task, with a focus on Pavlovian biases, and how these changes relate to changes in symptoms in people with depression receiving behavioral activation therapy versus a waitlist control. The findings support a relationship between decreases in anhedonia and reductions in Pavlovian bias. This is an interesting and well-written paper and the results have the potential to be highly impactful. Strengths include the use of a waitlist control and solid parameter recovery and reliability values for task performance.

1) The treatment group did show changes in the TEPS scale measuring anhedonia relative to the waitlist control, but other measures, including measures of depression severity (PHQ-9 and IDS) and behavioral activation-relevant symptoms (BADS) did not show changes. Is this degree of change consistent with the published literature for this treatment modality?

2) The a priori power analysis was for N=63 per group, but the 3-week assessment had only N=23 in the treatment group. Is it possible that null effects (and possibly significant findings: Loken, E. and Gelman, A. (2017). Measurement error and the replication crisis. Science 355, 584-585. DOI:10.1126/science.aal3618) are due to low power?

Reviewer #2: In the study by Berndt et al., the authors investigate the effect of behavioural activation therapy on Pavlovian biases in an affective go/nogo task as part of the UK NHS talking therapy service. They compare parameters fitted using reinforcement learning between an active and control group. In line with their hypothesis, they find that behavioural activation increases appetitive and reduces aversive Pavlovian biases. They also find that the effect of behavioural activation on anhedonia is mediated by this change in Pavlovian bias.

This is a rare gem of a study which applies ideas from computational psychiatry in a realistic treatment setting, bridging between mechanistic theory and patient symptoms. It is a heroic effort and a study that was clearly conducted very carefully. I enjoyed reading it and have a few questions and suggestions below.

Main finding of changes in Pavlovian bias (Fig4): While the study is very well written and the effects seem sound and convincing, explanations and visual illustrations of the data are relatively brief. To get a good grasp of the key effect, some additional analyses and plots would be very helpful and make the manuscript more convincing. For example, plotting the key result in an intuitive way by illustrating the effect of changing the affective bias in the computational model on some basic behavioural metric. This could maybe be achieved by plotting the tendency to produce a go action (% go) when Vt is <0 or >0, i.e. when the expected value was be positive or negative (for the two groups and two time points).

Relatedly, it seems that Figure S4E shows a trend-wise interaction driven by an effect in NoGo to win. It seems after behavioural activation, the active group is less accurate at nogo to win. Is this driven by the same effect captured by the computational modelling analyses, i.e. by expectation of reward making them go (when in this condition, they should not go), or is it due to some other mechanism?

Model fits (Fig4): While some models are visually clearly better than others in Figure 4B when overlaid on the data, even the winning model does not capture all trends and variations. Because there are no error bars around the actual data line (black line in Fig 4B), it is a bit difficult to see how consistent some of the deviations between model and data are across participants. Error bars, some more information on individual goodness of fit, and on what aspects of the data can consistently (?) not be explained well and why would help assess the goodness of the winning model. Also, some more descriptive statistics, such as the percentage of choices correctly explained (across groups, time points, and task conditions).

Behavioural activation therapy (Fig S2): For someone less familiar with the precise expected effects of behavioural activation at the time scale studied, can the behavioural activation therapy be seen as having been successful/effective if symptom changes are similar in the active and control groups across all measures but one (TEPS)? Is this expected? It would be helpful to comment and expand on this in the introduction or discussion.

Figure 5B, relationship TEPS and aversive bias: Was there a difference between groups? It seems that the correlation might be driven more by the control group and thus a change in TEPS not caused by behavioural activation? What are the authors’ thoughts on this? And how does this relate to the mediation analysis?

Reliability (Fig S5): It is great to see reliability analyses being conducted with so much care. The reliability of the aversive Pavlovian bias looks poorer than other parameters: this might be an illusion due to the plot axes not being symmetrical in Fig S5 which should be corrected but could also potentially be because only a handful of datapoints are driving the positive correlation for this parameter. Have the authors got any thoughts on this or looked into it further? Have they considered a robust regression analysis less driven by outliers? It is one of the key parameters for the paper, so its reliability seems important. I am aware that many studies still do not report test-retest reliability and that the reliability is overall good and I commend the authors for looking at this so carefully.

Minor

How is the combined Pavlovian bias defined?

It would be helpful to plot key results for the individual Pavlovian biases as well (appetitive and aversive, not combined) to show the direction of changes more clearly.

The introduction could be more specific about the precise RL deficits seen in depression, it is kept quite vague.

If all findings were hypothesised a priori, did the authors consider preregistering the study? If they did, please cite the repository.

Given the 130 minutes per time point could be completed within a 7-day window, more detail on actual completion times/spread should be added to the supplement, if possible.

The PHQ-9 asks about suicidal thoughts – were any procedures in place to offer additional support if a patient reported suicidal ideation?

Figure1: the colours in C are not clear/explained and boxes not aligned.

A few more sentences in the discussion expanding on how Pavlovian processes might be modified in depression and made a potential treatment target in the future would be helpful.

I suggest adding the computational model specifications to the main part of the manuscript, instead of the supplement.

**********

**Have the authors made all data and (if applicable) computational code underlying the findings in their manuscript fully available?**

The PLOS Data policy requires authors to make all data and code underlying the findings described in their manuscript fully available without restriction, with rare exception (please refer to the Data Availability Statement in the manuscript PDF file). The data and code should be provided as part of the manuscript or its supporting information, or deposited to a public repository. For example, in addition to summary statistics, the data points behind means, medians and variance measures should be available. If there are restrictions on publicly sharing data or code —e.g. participant privacy or use of data from a third party—those must be specified.

Reviewer #1: None

Reviewer #2: Yes

**********

PLOS authors have the option to publish the peer review history of their article (what does this mean?). If published, this will include your full peer review and any attached files.

If you choose “no”, your identity will remain anonymous but your review may still be made public.

**Do you want your identity to be public for this peer review?**  For information about this choice, including consent withdrawal, please see our Privacy Policy.

Reviewer #1: No

Reviewer #2: No

[NOTE: If reviewer comments were submitted as an attachment file, they will be attached to this email and accessible via the submission site. Please log into your account, locate the manuscript record, and check for the action link "View Attachments". If this link does not appear, there are no attachment files.]

**Figure resubmission:**

While revising your submission, we strongly recommend that you use PLOS’s NAAS tool (https://ngplosjournals.pagemajik.ai/artanalysis) to test your figure files. NAAS can convert your figure files to the TIFF file type and meet basic requirements (such as print size, resolution), or provide you with a report on issues that do not meet our requirements and that NAAS cannot fix.

After uploading your figures to PLOS’s NAAS tool - https://ngplosjournals.pagemajik.ai/artanalysis, NAAS will process the files provided and display the results in the "Uploaded Files" section of the page as the processing is complete. If the uploaded figures meet our requirements (or NAAS is able to fix the files to meet our requirements), the figure will be marked as "fixed" above. If NAAS is unable to fix the files, a red "failed" label will appear above. When NAAS has confirmed that the figure files meet our requirements, please download the file via the download option, and include these NAAS processed figure files when submitting your revised manuscript.

**Reproducibility:**

To enhance the reproducibility of your results, we recommend that authors of applicable studies deposit laboratory protocols in protocols.io, where a protocol can be assigned its own identifier (DOI) such that it can be cited independently in the future. Additionally, PLOS ONE offers an option to publish peer-reviewed clinical study protocols. Read more information on sharing protocols at https://plos.org/protocols?utm_medium=editorial-email&utm_source=authorletters&utm_campaign=protocols

# Author comment: Author response to Decision Letter 1

**DOI:** 10.1371/journal.pcbi.1014439.r002

11 Mar 2026

Attachment Submitted filename: Response to Reviewers.pdf

# Peer review: Decision Letter 1

**DOI:** 10.1371/journal.pcbi.1014439.r003

**Authors:** Marieke Karlijn van Vugt

1 May 2026

PCOMPBIOL-D-25-02123R1

Early reduction in aversive Pavlovian bias as a mediator of anhedonia improvement during Behavioural Activation in realistic treatment settings

PLOS Computational Biology

Dear Dr. Berndt,

Thank you for submitting your manuscript to PLOS Computational Biology. After careful consideration, we feel that it has merit but does not fully meet PLOS Computational Biology's publication criteria as it currently stands. Therefore, we invite you to submit a revised version of the manuscript that addresses the points raised during the review process.

Please submit your revised manuscript by Jul 01 2026 11:59PM. If you will need more time than this to complete your revisions, please reply to this message or contact the journal office at ploscompbiol@plos.org. When you're ready to submit your revision, log on to https://www.editorialmanager.com/pcompbiol/ and select the 'Submissions Needing Revision' folder to locate your manuscript file.

Please include the following items when submitting your revised manuscript:

* A letter that responds to each point raised by the editor and reviewer(s). You should upload this letter as a separate file labeled 'Response to Reviewers'. This file does not need to include responses to formatting updates and technical items listed in the 'Journal Requirements' section below.

* A marked-up copy of your manuscript that highlights changes made to the original version. You should upload this as a separate file labeled 'Revised Manuscript with Track Changes'.

* An unmarked version of your revised paper without tracked changes. You should upload this as a separate file labeled 'Manuscript'.

If you would like to make changes to your financial disclosure, competing interests statement, or data availability statement, please make these updates within the submission form at the time of resubmission. Guidelines for resubmitting your figure files are available below the reviewer comments at the end of this letter.

We look forward to receiving your revised manuscript.

Kind regards,

Marieke Karlijn van Vugt, PhD

Section Editor

PLOS Computational Biology

Marieke van Vugt

Section Editor

PLOS Computational Biology

**Additional Editor Comments:**

Thank you for making substantial revisions. However, one of the reviewers still has some remaining concerns about how the data are communicated, so I am sending this back to you for minor revisions.

**Reviewers' comments:**

Reviewer's Responses to Questions

**Comments to the Authors:**

**Please note here if the review is uploaded as an attachment.**

Reviewer #2: Thank you the work you put into revising the manuscript, which I believe helped increase the clarity and robustness of what was already a very interesting report. I have only one small comment left:

In response to my first point, the authors added Figure S7 which is a theoretical depiction of how different parameters in the model map onto different behaviours. This is helpful and would predict strong effects on Nogo-to-win and Go-to-avoid-loss trials. Apologies if my initial comment was confusing, but I was actually asking for the actual data to be plotted in an intuitive way so that readers could understand the change in the combined model bias parameters induced by BA therapy in a way that is intuitive and easy to follow. Maybe this is not possible, and of course the raw data leading to these results are shown in Figure 3C, but it takes a while for the reader to link the computational effect shown in Fig 5A to the raw data in 3C and to their key message. This could be made easier for the reader and thus potentially mean more people immediately understand what the authors are trying to convey, making the manuscript more accessible. For example, if indeed the appetitive bias has increased and the aversive bias has diminished, then the prediction would be that the largest effects are on the purple and green Nogo-to-win and Go-to-avoid-loss conditions (in active vs control). It seems BA-mediated effects are particularly pronounced (visually) in the no-go-to-win. Is it fair to conclude then that the effect on the combined parameter Δ combined is more/mostly/predominantly driven by NoGo to win and reflects an increased appetitive Pavlovian bias more than a reduced aversive bias? I acknowledge that (in response to my point 2) the authors refrain from interpreting this effect (which is similarly reflected in Fig S6E) because it is not significant in a triple interaction of Time x Group x Condition. However, it seems if the model predicts changes particularly in NoGo to Avoid Loss or NoGo to Win, and the combined bias change is significant, a post-hoc test focusing on these two conditions would be justified to fully portray and explain the aspects of the data that drive the model-derived combined bias effect. I think this would be an interesting addition as the reader is left wondering whether the Pavlovian bias change was similar in the appetitive and aversive domains which does not seem to be the case (and I wasn’t sure if it squares well with this statement in the discussion “This would suggest that individuals with stronger aversive Pavlovian bias at baseline may benefit most from BA”.). Thanks for considering this. I would leave it up to the authors to consider making this change.

**********

**Have the authors made all data and (if applicable) computational code underlying the findings in their manuscript fully available?**

The PLOS Data policy requires authors to make all data and code underlying the findings described in their manuscript fully available without restriction, with rare exception (please refer to the Data Availability Statement in the manuscript PDF file). The data and code should be provided as part of the manuscript or its supporting information, or deposited to a public repository. For example, in addition to summary statistics, the data points behind means, medians and variance measures should be available. If there are restrictions on publicly sharing data or code —e.g. participant privacy or use of data from a third party—those must be specified.

Reviewer #2: Yes

**********

PLOS authors have the option to publish the peer review history of their article (what does this mean?). If published, this will include your full peer review and any attached files.

If you choose “no”, your identity will remain anonymous but your review may still be made public.

**Do you want your identity to be public for this peer review?**  For information about this choice, including consent withdrawal, please see our Privacy Policy.

Reviewer #2: No

[NOTE: If reviewer comments were submitted as an attachment file, they will be attached to this email and accessible via the submission site. Please log into your account, locate the manuscript record, and check for the action link "View Attachments". If this link does not appear, there are no attachment files.]

**Figure resubmission:**

-->While revising your submission, we strongly recommend that you use PLOS’s NAAS tool (https://ngplosjournals.pagemajik.ai/artanalysis) to test your figure files. NAAS can convert your figure files to the TIFF file type and meet basic requirements (such as print size, resolution), or provide you with a report on issues that do not meet our requirements and that NAAS cannot fix.

After uploading your figures to PLOS’s NAAS tool - https://ngplosjournals.pagemajik.ai/artanalysis, NAAS will process the files provided and display the results in the "Uploaded Files" section of the page as the processing is complete. If the uploaded figures meet our requirements (or NAAS is able to fix the files to meet our requirements), the figure will be marked as "fixed" above. If NAAS is unable to fix the files, a red "failed" label will appear above. When NAAS has confirmed that the figure files meet our requirements, please download the file via the download option, and include these NAAS processed figure files when submitting your revised manuscript.

**Reproducibility:**

To enhance the reproducibility of your results, we recommend that authors of applicable studies deposit laboratory protocols in protocols.io, where a protocol can be assigned its own identifier (DOI) such that it can be cited independently in the future. Additionally, PLOS ONE offers an option to publish peer-reviewed clinical study protocols. Read more information on sharing protocols at https://plos.org/protocols?utm_medium=editorial-email&utm_source=authorletters&utm_campaign=protocols

# Author comment: Author response to Decision Letter 2

**DOI:** 10.1371/journal.pcbi.1014439.r004

13 May 2026

Attachment Submitted filename: PLOSCB_resp_v3.pdf

# Peer review: Decision Letter 2

**DOI:** 10.1371/journal.pcbi.1014439.r005

**Authors:** Marieke Karlijn van Vugt, Varun Dutt

14 Jun 2026

Dear Ms Berndt,

We are pleased to inform you that your manuscript 'Early reduction in aversive Pavlovian bias as a mediator of anhedonia improvement during Behavioural Activation in realistic treatment settings' has been provisionally accepted for publication in PLOS Computational Biology.

Before your manuscript can be formally accepted you will need to complete some formatting changes, which you will receive in a follow up email. A member of our team will be in touch with a set of requests.

Please note that your manuscript will not be scheduled for publication until you have made the required changes, so a swift response is appreciated.

IMPORTANT: The editorial review process is now complete. PLOS will only permit corrections to spelling, formatting or significant scientific errors from this point onwards. Requests for major changes, or any which affect the scientific understanding of your work, will cause delays to the publication date of your manuscript.

Should you, your institution's press office or the journal office choose to press release your paper, you will automatically be opted out of early publication. We ask that you notify us now if you or your institution is planning to press release the article. All press must be co-ordinated with PLOS.

Thank you again for supporting Open Access publishing; we are looking forward to publishing your work in PLOS Computational Biology.

Best regards,

Varun Dutt, Ph.D

Academic Editor

PLOS Computational Biology

Marieke van Vugt

Section Editor

PLOS Computational Biology

***********************************************************

Dear Authors,

Thank you for submitting the revised version of your manuscript entitled “Early reduction in aversive Pavlovian bias as a mediator of anhedonia improvement during Behavioural Activation in realistic treatment settings.”

I have reviewed the revised manuscript, the response letter, and the reviewer’s comments. The remaining concern from the previous round related primarily to the clarity and interpretation of Figure S7 and how the model-derived Pavlovian bias effects relate to the observed behavioural data. The authors have now addressed this issue satisfactorily by revising Figure S7, clarifying the relevant analysis populations, adding post-hoc behavioural tests for the key incongruent conditions, and appropriately avoiding overinterpretation where condition-level effects were not statistically significant.

The manuscript is now clearer, better contextualized, and more accessible to readers. The revisions improve the link between the computational model and the behavioural results, while preserving appropriate caution in interpretation. The reviewer has indicated that all comments have been addressed and recommends acceptance.

Overall, the manuscript makes a valuable contribution by connecting computational measures of Pavlovian bias with clinically meaningful improvement during Behavioural Activation in realistic treatment settings. I am satisfied that the authors have addressed the outstanding concerns.

I am pleased to recommend the manuscript for acceptance in PLOS Computational Biology.

Sincerely,

Prof. Varun Dutt

Academic Editor

Reviewer's Responses to Questions

**Comments to the Authors:**

**Please note here if the review is uploaded as an attachment.**

Reviewer #2: Thank you for clarifying the results show in Figure S7 - and for your great work on improving the manuscript. All my comments have been addressed and I congratulate the authors on a really nice piece of work.

**********

**Have the authors made all data and (if applicable) computational code underlying the findings in their manuscript fully available?**

The PLOS Data policy requires authors to make all data and code underlying the findings described in their manuscript fully available without restriction, with rare exception (please refer to the Data Availability Statement in the manuscript PDF file). The data and code should be provided as part of the manuscript or its supporting information, or deposited to a public repository. For example, in addition to summary statistics, the data points behind means, medians and variance measures should be available. If there are restrictions on publicly sharing data or code —e.g. participant privacy or use of data from a third party—those must be specified.

Reviewer #2: Yes

**********

PLOS authors have the option to publish the peer review history of their article (what does this mean?). If published, this will include your full peer review and any attached files.

If you choose “no”, your identity will remain anonymous but your review may still be made public.

**Do you want your identity to be public for this peer review?**  For information about this choice, including consent withdrawal, please see our Privacy Policy.

Reviewer #2: No

# Editor report: Acceptance letter

**DOI:** 10.1371/journal.pcbi.1014439.r006

**Authors:** Marieke Karlijn van Vugt, Varun Dutt

PCOMPBIOL-D-25-02123R2

Early reduction in aversive Pavlovian bias as a mediator of anhedonia improvement during Behavioural Activation in realistic treatment settings

Dear Dr Berndt,

I am pleased to inform you that your manuscript has been formally accepted for publication in PLOS Computational Biology. Your manuscript is now with our production department and you will be notified of the publication date in due course.

The corresponding author will soon be receiving a typeset proof for review, to ensure errors have not been introduced during production. Please review the PDF proof of your manuscript carefully, as this is the last chance to correct any errors. Please note that major changes, or those which affect the scientific understanding of the work, will likely cause delays to the publication date of your manuscript.

Soon after your final files are uploaded, unless you have opted out, the early version of your manuscript will be published online. The date of the early version will be your article's publication date. The final article will be published to the same URL, and all versions of the paper will be accessible to readers.

For Research, Software, and Methods articles, you will receive an invoice from PLOS for your publication fee after your manuscript has reached the completed accept phase. If you receive an email requesting payment before acceptance or for any other service, this may be a phishing scheme. Learn how to identify phishing emails and protect your accounts at https://explore.plos.org/phishing.

Thank you again for supporting PLOS Computational Biology and open-access publishing. We are looking forward to publishing your work!

With kind regards,

Anitha Samidurai

PLOS Computational Biology | Carlyle House, Carlyle Road, Cambridge CB4 3DN | United Kingdom ploscompbiol@plos.org | Phone +44 (0) 1223-442824 | ploscompbiol.org | @PLOSCompBiol
